# Novel Binary Mixtures of Alkanolamine Based Deep Eutectic Solvents with Water—Thermodynamic Calculation and Correlation of Crucial Physicochemical Properties

**DOI:** 10.3390/molecules27030788

**Published:** 2022-01-25

**Authors:** Bartosz Nowosielski, Marzena Jamrógiewicz, Justyna Łuczak, Dorota Warmińska

**Affiliations:** 1 Department of Physical Chemistry, Faculty of Chemistry, Gdańsk University of Technology, ul. Narutowicza 11/12, 80-233 Gdańsk, Poland; bartosz.nowosielski@pg.edu.pl; 2 Department of Physical Chemistry, Faculty of Pharmacy, Medical University of Gdańsk, Al. Gen. Hallera 107, 80-416 Gdańsk, Poland; marzena.jamrogiewicz@gumed.edu.pl; 3 Department of Process Engineering and Chemical Technology, Faculty of Chemistry, Gdańsk University of Technology, ul. Narutowicza 11/12, 80-233 Gdańsk, Poland; justyna.luczak@pg.edu.pl

**Keywords:** DES, deep eutectic solvents, aqueous mixtures, excess properties, JAM, PFP

## Abstract

This paper demonstrates the assessment of physicochemical and thermodynamic properties of aqueous solutions of novel deep eutectic solvent (DES) built of tetrabutylammonium chloride and 3-amino-1-propanol or tetrabutylammonium bromide and 3-amino-1-propanol or 2-(methylamino)ethanol or 2-(butylamino)ethanol. Densities, speeds of sound, refractive indices, and viscosities for both pure and aqueous mixtures of DES were investigated over the entire range of compositions at atmospheric pressure and T = (293.15 ‒ 313.15) K. It was concluded that the experimental data were successfully fitted using the Jouyban–Acree model with respect to the concentration. Obtained results showed that this mathematical equation is an accurate correlation for the prediction of aqueous DES properties. Key physicochemical properties of the mixtures—such as excess molar volumes, excess isentropic compressibilities, deviations in viscosity, and deviations in refractive indices—were calculated and correlated by the Redlich–Kister equation with temperature-dependent parameters. The non-ideal behavior of the studied systems were also evaluated by using the Prigogine−Flory−Patterson theory and the results were interpreted in terms of interactions between the mixture components.

## 1. Introduction

Deep eutectic solvents (DES) are very important and well-known components or materials used often in chemistry but also in other industries as pharmacy, chemical technology as an inexpensive solvent/component being sustainable alternative to the conventional organic solvents which are non-ecological. Currently, the advancement of different DES had enabled the production of materials for unique purpose for reaction medium, biodiesel processes, metal electrodeposition, nanotechnology, and others [1]. Global research is still focused on the developing new innovative DES which will probably replace non-ecological classic solvents as well [2,3].

Deep eutectic solvents, based on urea and quaternary ammonium salts, were first reported by Abbott et al. [4]. Since then many derivatives have been invented and applied. Generally, DESs are built from hydrogen bond acceptor (HBA) and hydrogen bond donor (HBD) in the appropriate molar ratio forming complexes through hydrogen bonds. As a result, deep eutectic solvents have a lower melting point than their components [5].

DESs share many properties with room temperature ionic liquid (RTIL). They are practically non-volatile and non-flammable, and exhibit high thermal and electro-chemical stability, but are definitely cheaper, less toxic, and often biodegradable in comparison with RTIL [6]. Additionally, since the physical properties of DES are dependent on the composition and proportions of the components making up a given eutectic mixture, it is possible to propose a particular composition whose properties should be applied specifically [1,7].

Deep eutectic solvents are highly hygroscopic liquids, so trace amounts of water are often unavoidable as impurity [8]. However, depending on the application, water can be deliberately added to the DES to modulate the physicochemical properties of the solvent, especially mass transfer properties such as viscosity.

Aqueous mixtures of DES have already been used in nanoparticle synthesis [9], carbon dioxide absorption [10,11], and as media in chemical reactions [12]. Some conflicting information is found in the literature regarding the impact of water in DES on processes of CO_2_ absorption. Su et al. [10] discovered that even a small addition of water to DES composed of choline chloride (ChCl) and urea reduces the solubility of carbon dioxide, while Li et al. [11] concluded that the solubility of CO_2_ increases in tetramethylammonium chloride and monoethanolamine DES after the addition of H_2_O, and reaches a maximum at 10% water content. Therefore, since the water content has a great influence on the physicochemical properties of deep eutectic solvents, it is crucial to control it in aqueous DES solutions from a technological point of view and in further application. A significant part of the research carried out so far has been devoted to the properties of pseudo-pure DES [13,14,15,16]. Thermodynamic properties of aqueous DESs are also rarely published [17]. Kuddushi et al. measured densities of aqueous solution of DES composed of ChCl with glutaric acid and malonic acid [18]. They calculated excess molar volume ($V_{m}^{E}$) for those systems that found to be negative, indicating strong interactions between water and DES molecules. Additionally, volumetric properties were described for such aqueous DES systems as: allyltriphenyl phosphonium bromide (ATPPB) with diethylene glycol (DEG) [19] or triethylene glycol (TEG) [20], ChCl with ethylene glycol or glycerol [21] and ChCl with lactic acid [22].

Studies that consider other properties of aqueous DES solutions—such as acoustic properties [18], refractive indices [21,23], and viscosities [23,24]—are still rare and not popular. All of them confirm that the interactions between DES and water molecules significantly influence on thermodynamic properties of aqueous DES solutions.

In general, most of the research that has been carried out to date on deep eutectic solvents as potential CO_2_ absorbers are devoted to DESs in which physical absorption of carbon dioxide occurs. The carbon dioxide absorption capacity in those DESs is lower than that in commercially used absorbers therefore their potential application in industry is significantly limited. Therefore, the aim of this work is to characterize and better understand the water solutions of deep eutectic solvents based on alkanolamines with chemical absorption capacity in terms of their suitability for the effective separation of carbon dioxide from gas streams at relatively low pressure. It is known from the literature that carbon dioxide capacity, apart from others factors, depends also on the strength of intermolecular interactions between DES components [25,26]. As the interactions between HBA and HBD increase, the CO_2_ solubility decreases. Similar effects can be expected for aqueous solutions of DESs, where increasing strength of the DES-H2O interactions might result in a decrease of carbon dioxide capacity due to the weaker interactions between CO_2_ and DES [27]. The final effect of the presence of water in DES on the solubility of CO_2_ will also depend on which of the components (DES or water) will have the greater affinity for the gas absorbed. Herein, we prepared deep eutectic solvents built of tetrabutylammonium bromide with 3-amino-1-propanol (AP), 2-(methylamino)ethanol (MAE), or 2-(butylamino)ethanol (BAE) and tetrabutylammonium chloride (TBAC) with AP, at 1:6 molar ratios. Then, the physicochemical properties of pure and aqueous solutions of DESs as density, speed of sound, viscosity, and refractive index were measured, and Jouyban–Acree predictive model (JAM) was used to correlate the experimental data. Several mathematical models for the correlation of physical properties of binary mixtures can be found in the literature [28]. However, these models were used mainly to correlate the density of mixtures. Thus, we decided to use the JAM equation, as so far it has also been used to predict the viscosity of two-component mixtures of classical solvents [29]. Thermodynamic excess properties—including excess molar volume, excess isobaric thermal expansion, excess isentropic compressibility, deviation in refractive indices, deviation in viscosities, and excess Gibbs energy of activation for viscous flow—were calculated and correlated by the Redlich–Kister-type polynomial equation considered as the most common and accurate mathematical model for this purpose. Prigogine–Flory–Patterson Theory (PFP) was used to correlate the experimental excess molar volume as the most accepted theory to interpret the behavior of non-ideal liquid solution, which has been applied to many mixtures of classical solvents as well as to systems containing ionic liquids and some aqueous solutions of DESs. The effect of the HBA anion type of obtained DES solutions was evaluated as well as the order and length of the alkyl chain of each alkanolamine were discussed. The influence of temperature on thermodynamic properties of DES solutions was also involved in this study.

## 2. Materials and Methods

### 2.1. Chemicals

The chemicals used in this study—3-amino-1-propanol (AP), 2-(methylamino)ethanol (MAE), 2-(butylamino)ethanol (BEA), tetrabutylammonium bromide (TBAB), and tetrabutylammonium chloride (TBAC)—were purchased from Sigma-Aldrich. TBAC was purified by double crystallization from acetone by adding diethyl ether. All salts were dried under reduced pressure before use, TBAB at 323 K for 48 h while TBAC at 298.15 K for several days. The corresponding information and the chemical structures of the DESs components are presented in Table 1.

### 2.2. Preparation of DESs and Their Aqueous Solutions

DESs were prepared by mass with the same molar ratio of 1:6 salt to amino alcohol. The weighing was done using an analytical balance (Mettler Toledo) with the precision of 0.1 mg. The standard uncertainty in the mass fraction was estimated to be less than ±1 × 10^−4^. The combinations of the quaternary ammonium salts and AP/MAE/BEA were mixed at 353.15 K for 1 h using a magnetic stirrer in a fume hood until a homogeneous and uniform liquid without any precipitate was formed. Water content of DESs was measured using a Mettler Toledo Coulometric Karl–Fischer titrator (899 Coulometer apparatus from Metrohm) and it was found to be less than 0.0016 mass fraction. Table 2 displays the abbreviation of chemicals and DESs along with their molar mass, molar ratio, mass fraction, and water content.

Deionized, double distilled, degassed water with a specific conductance of $1.15\times 10^{-6}\;S\cdot {\mathrm{cm}}^{-1}$ was used for the preparation of aqueous mixtures of DESs. The water contents in DES was accounted for upon solution preparation.

### 2.3. Physical Properties Measurements

#### 2.3.1. Density and Speed of Sound

Measurements of density and speed of sound were performed by using a digital vibration-tube analyzer (Anton Paar DSA 5000, Graz, Austria) with proportional temperature control that kept the samples at working temperature with an accuracy of 0.01 K. Experimental frequency for the measurements of the ultrasonic speed was equal to 3 MHz. The apparatus was calibrated with double distilled deionized and degassed water and dry air at atmospheric pressure according to the apparatus catalog procedure. The experimental uncertainty of density and ultrasonic velocity measurements was better than 35 × 10^−3^ kg m^−3^ and 2 × 10^−1^ m s^−1^, respectively.

#### 2.3.2. Viscosity

Viscosities of the solvents were determined using LVDV-III Programmable Rheometer (cone-plate viscometer; Brookfield Engineering Laboratory, USA), controlled by a computer. The temperature of the samples was controlled within ± 0.01K using a thermostatic water bath (PolyScience 9106, Niles, IL, USA). The display of the viscosimeter was verified with certified viscosity standard N100 and S3 provided by Cannon at 298.15 ± 0.01 K. The standard uncertainty of viscosity measurement was better than 1%.

#### 2.3.3. Refractive Index

The refractive indices were measured using an Abbe refractometer (RL-3, Warsaw, Poland) equipped with a thermostat for controlling the cell temperature with an accuracy of ±0.1 K. The standard uncertainty of refractive index measurement on the n_D_ scale was 0.0002. At least three independent measurements were taken for each sample at each temperature to assure reproducibility of the measurement.

#### 2.3.4. Isobaric Heat Capacity

A Mettler Toledo Star One differential scanning calorimeter (DSC), STAR-1 System (Mettler Toledo, Greifensee, Switzerland), was used to measure specific heat capacities of novel DESs. The DSC instrument was calibrated by the indium standard prior to sample measurements. During the measurement, an inert atmosphere was created under a nitrogen flow of 60 mL min^−1^. The sapphire method for cp determination was used [30]. A ‘baseline’ or blank measurement was performed for heating rate 10 K min^−1^. All of the results obtained were blank curve corrected and performed twice. The test material and the reference were placed into individual aluminium crucibles which were then sealed with pierced lids. The data from the DSC were recorded and then analyzed to obtain the *Cp* from the data.

## 3. Results and Discussion

### 3.1. Physical Properties of Binary Mixtures

**Figure 1:**
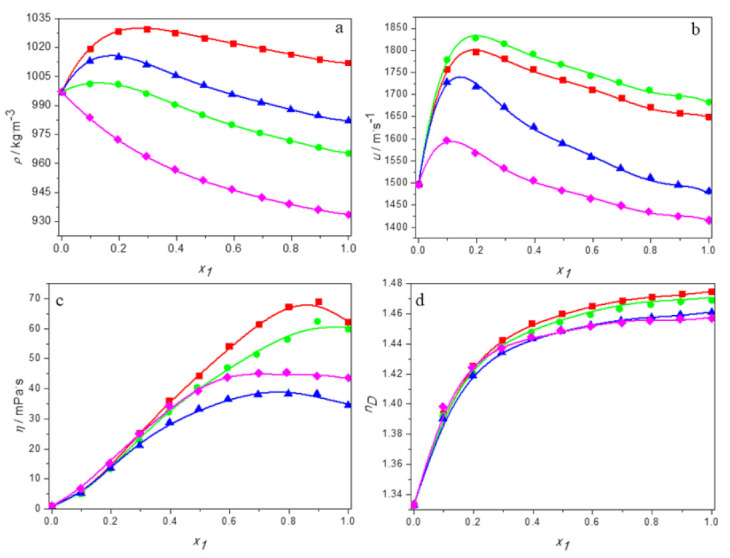
The dependence of the physical properties of aqueous solutions of DESs on molar fraction of deep eutectic solvent at 298.15K: (**a**) density; (**b**) speed of sound; (**c**) viscosity; (**d**) refractive index. ■ DES1; ● DES2; ▲ DES3; ♦ DES4; —, Equation (1).

The experimental values of density, speed of sound, viscosity, and refractive indices for aqueous solutions of DESs consisting of tetrabutylammonium bromide and 3-amino-1-propanol or 2-(methylamino)ethanol or 2-(butylamino)ethanol and for the aqueous solutions of DES built of tetrabutylammonium chloride and 3-amino-1-propanol over the entire range of compositions at temperatures ranging from 293.15 K to 313.15 K are reported in Tables S1–S4. Moreover, in Figure 1 the physical properties are plotted as a function of the DES molar fraction at 298.15 K for all systems studied. As it can be observed, depending on the properties, its dependence on the deep eutectic solvent content varies. Moreover, all trends are nonlinear, indicating deviation from the ideal course.

Taking into account the density, its values decrease in the whole range of DES concentrations only for aqueous TBAB:BAE (DES4) solutions for which a negative deviation from ideal behavior is observed. For the other systems, the density increases with increasing DES concentration at low deep eutectic solvent content, reaches its maximum value at certain molar fraction of DES, and afterwards begins to decrease. The composition of the solution with the highest density depends on both the amino alcohol and the salt.

The dependence of the sound velocity and viscosity of aqueous DES solutions on the molar fraction of DES also shows a maximum. However, in the case of viscosity, unlike the speed of sound and the density, it occurs at high DES content. The refractive index increases monotonically in the whole range of DES concentrations for all systems.

When the temperature dependence is considered, one can observe that all properties decrease with the increase of temperature as the result of thermal expansion.

The experimental values of of density, speed of sound, viscosity, and refractive indices of binary mixtures were correlated by using of Jouyban–Acree model [29,31,32,33]. This mathematical model uses the physicochemical properties of the individual solvents as input data and a number of curve-fitting parameters represent the effects of solvent–solvent interactions in the solution

(1) $$lny=x_{1}lny_{1}+x_{2}lny_{2}+\frac{x_{1}x_{2}}{T}\overset{i=n}{\underset{i=0}{\sum }}J_{i}{\left(x_{1}-x_{2}\right)}^{2}$$

The *y*, *y*_1_, and *y*_2_ are the physical properties of the mixture, deep eutectic solvent and water, at specific temperature. The *x*_1_ and *x*_2_ are mole fractions of DES and water, respectively. The *J_i_* terms are coefficients of the model computed by using a zero-intercept regression analysis

(2) $$lny-x_{1}lny_{1}-x_{2}lny_{2}=\frac{x_{1}x_{2}}{T}\overset{i=n}{\underset{i=0}{\sum }}J_{i}{\left(x_{1}-x_{2}\right)}^{2}$$

Root mean square deviation of fit (RMSD) and the average deviation (ARD %) were calculated according to the following equations

(3) $$RMSD={\left[\frac{\sum {\left(Y_{exp}-Y_{pred}\right)}^{2}}{n-k}\right]}^{1/2}$$

(4) $$ARD\;\%=\frac{100}{n}\sum \frac{\left|y_{exp}-y_{pred}\right|}{y_{pred}}$$

where *n* is the number of experimental data, *k* is the number of parameters of model and *Y* is equal to *lny*.

Parameters *J_i_* of Equation (1), root mean square deviation of fit (RMSD) and the corresponding average relative deviation (ARD %) for the systems studied at *T* = (293.15 to 313.15) K are presented in Table S5 (Supplementary Materials).

Moreover, the values of density, speed of sound, viscosity, and refractive index obtained by JAM are depicted as the smoothed solid lines in Figure 1. As can be seen, the Jouyban–Acree model correlates the experimental physical properties satisfactorily, especially for density and refractive index, for which average relative deviations are the same order as the experimental uncertainty. Thus, the JAM can be considered a reliable model for predicting the densities and refractive indices as well as it can be used for estimation of the speeds of sound and viscosity of aqueous DES solutions, for which, however, higher ARD % are observed.

### 3.2. Volumetric Properties

#### 3.2.1. Excess Molar Volume

The excess molar volumes (*V^E^*) were calculated using the experimental density data according to the following equation

(5) $$V^{E}=\frac{x_{1}M_{1}+x_{2}M_{2}}{\rho }-\frac{x_{1}M_{1}}{\rho_{1}}-\frac{x_{2}M_{2}}{\rho_{2}}$$

where *d* is the density of the mixture and *x_i_, M_i_*, and *ρ_i_* are: the mole fraction, the molar mass and density of DES (*i* = 1) and water (*i* = 2), respectively.

**Figure 2:**
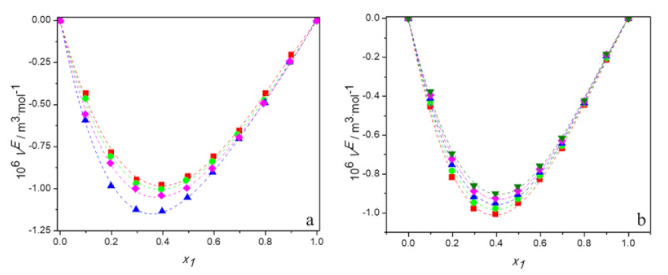
Dependence of the excess molar volume of aqueous solutions of DESs on molar fraction of deep eutectic solvent: (**a**) at 298.15 K for ■ DES1; ● DES2; ▲ DES3; ♦ DES4; (**b**) for DES1 at 298.15 K (■); 298.15 K (●); 303.15 K (▲); 308.15 K (♦); 313.15 K (▼); —, Equation (6).

The obtained values of *V^E^* are presented in Tables S1–S4 and plotted as a function of the DES mole fraction, *x*_1_, in Figure 2. Figure 2a shows the plots of *V^E^* against mole fraction of DES for all studied mixtures at 298.15 K and Figure 2b depicts the temperature dependence of excess molar volumes for the system (TBAB:BAE + water) as an example. It can be observed in these figures that the curves of *V^E^* are asymmetrical and their values are negative over the whole composition and temperature range. The minimum was found between mol fraction of 0.35 and 0.4 of DES equal to −0.98 for DES1, −1.00 for DES2, −1.13 for DES3, −1.02 for DES4. The asymmetry of the curves is due to the difference between the molar volumes of the components mixture.

In Figure 2, the dashed lines represent the correlated values according to the Redlich–Kister polynomial [34]

(6) $$V^{E}=x_{1}x_{2}\overset{2}{\underset{i=0}{\sum }}A_{i}{\left(x_{1}-x_{2}\right)}^{i}$$

where the *A_i_* values are adjustable parameters.

As it can be seen, the calculated values agree very well with the experimental data. The *A_i_* values were determined using the least squares method and they are listed in Tables S6–S9, along with their RMSD. For all systems, excess molar volumes were correlated using three-parameter Redlich–Kister polynomial equation.

The negative values of excess molar volumes can be explained based on the strength of the specific interactions, size, and shape of molecules. When DES is added to water, the intra-molecular interactions between DES or water molecules are disrupted and new hydrogen bonding interactions between water and chloride/bromide anion of HBA and between water and -OH group and -NH_2_ group or –NH of amino alcohol are forming. Moreover, water molecules—as much smaller than the deep eutectic solvent one—may fit into the interstices of the DES. Therefore, the filling effect of water in the interstices of DES, and the strong hydrogen bonding interactions between the unlike components of the systems, all lead to the negative values of the excess molar volumes.

The temperature dependence of the excess molar volumes can determine what kind of effect—i.e., the packing phenomenon or the strong forces between the components—is responsible for the negative values of *V^E^.* In general, as temperature increases, the specific interactions break down and due to the increased thermal fluctuation, more holes of sufficient size for the accommodation of the unlike component are formed. These effects influence the excess molar volume in a reverse manner. A decrease of specific interactions causes an increase in *V^E^* values, while a loosening of the DES structure leads to a decrease of excess molar volume with temperature. Thus, the observed increase of *V^E^* with rising temperature for all systems investigated suggests that specific interactions determine the volumetric behavior of aqueous solutions of deep eutectic solvents based on alcohol amine. A similar phenomenon was observed by other researchers for aqueous solutions of DES based on choline chloride [18,21,22,24,35,36,37,38] or allyltriphenylphosphonium bromide [19,20]. What is interesting, for the (DES + alcohol) systems, due to the decrease in the excess molar volume with temperature, the dominance of the packing effect was postulated [39,40].

The dominance of specific interactions in the aqueous solutions of the DESs studied can be confirmed by the Prigogine–Flory–Patterson (PFP) theory [41,42,43,44,45]. This theory has been originally used in interpreting the values of the excess molar volumes of binary systems formed by polar compounds which do not form strong electrostatic or hydrogen bond interactions. Over time, however, it has emerged that the use of the Flory formalism can still provide an interesting correlation between the excess volumes of more complex mixtures. So far, the PFP theory has been successfully applied to predict and model the excess molar volumes of many mixtures containing ionic liquids [46,47,48,49,50] and some aqueous systems with deep eutectic solvents [18,51,52].

According to the PFP theory, the excess molar volume contains three contributions: an interactional contribution, a free volume contribution, and a pressure contribution. The expression for *V^E^* is given as

(7) $$\frac{V^{E}}{x_{1}V_{1}^{*}+x_{2}V_{2}^{*}}=\frac{\left({\tilde{V}}^{1/3}-1\right){\tilde{V}}^{2/3}\psi_{1}\Theta_{2}\chi_{12}}{\left[\left(4/3\right){\tilde{V}}^{-1/3}-1\right]P_{1}^{*}}+\frac{-{\left({\tilde{V}}_{1}-{\tilde{V}}_{2}\right)}^{2}\left[\left(14/9\right){\tilde{V}}^{-1/3}-1\right]\psi_{1}\psi_{2}}{\left[\left(4/3\right){\tilde{V}}^{-1/3}-1\right]\tilde{V}}+\frac{\left({\tilde{V}}_{1}-{\tilde{V}}_{2}\right)\left(P_{1}^{*}-P_{2}^{*}\right)\psi_{1}\psi_{2}}{P_{2}^{*}\psi_{1}+P_{1}^{*}\psi_{2}}$$

where *V^E^* is excess molar volume, *x* mole fraction, V* characteristic volume, P* characteristic pressure, *ψ* molecular contact energy fraction, *θ* molecular surface fraction, $\tilde{V}$ reduced volume, and *χ*_12_ interactional parameter.

The reduced volume for pure substance *i* is defined in terms of the thermal expansion coefficients, *α_i_*, as

(8) $${\tilde{V}}_{i}={\left(\frac{1+\frac{4}{3}\alpha_{i}T}{1+\alpha_{i}T}\right)}^{3}$$

The reduced volume of mixture, $\tilde{V}$, is calculated from

(9) $$\tilde{V}=\psi_{1}\tilde{V_{1}}+\psi_{2}\tilde{V_{2}}$$

where the molecular contact energy fraction, *ψ*, is expressed by: $\psi_{1}=1-\psi_{2}=\frac{\phi_{1}p_{1}^{*}}{\phi_{1}p_{1}^{*}+\phi_{2}p_{2}^{*}}$ with the hardcore volume fraction, ϕ, calculated from $\phi_{1}=1-\phi_{2}=\frac{x_{1}V_{1}^{*}}{x_{1}V_{1}^{*}+x_{2}V_{2}^{*}}$.

The characteristic volume, $V_{i}^{*}$, is calculated from the molar volume from the expression $V_{i}^{*}=\frac{V_{i}^{0}}{\tilde{V_{i}}}$ and the characteristic pressure is expressed by

(10) $$p_{i}^{*}=\frac{\alpha_{i}}{\kappa_{Ti}}T{\tilde{V_{i}}}^{2}$$

where $\kappa_{Ti}$ is the isothermal compressibility obtained from the isentropic compressibility from the thermodynamic relation

(11) $$\kappa_{Ti}=\kappa_{Si}+\frac{V_{i}^{0}\alpha_{i}^{2}T}{C_{pi}}$$

with the isobaric heat capacity, $C_{pi}$.

The molecular surface fraction of component 2 is given by: $\Theta_{2}=\frac{\phi_{2}}{\phi_{1}\frac{s_{1}}{s_{2}}+\phi_{2}}$, in which the ratio of the surface contact sites per segment is given by

(12) s1s2=v2*v1*13

In present study, the thermal expansion coefficient, *α_p_*, defined as: $\alpha_{p}=\frac{1}{V}{\left(\frac{\partial V}{\partial T}\right)}_{P}$, was calculated by use the temperature dependence of density, which was found to be the second-order polynomial equation.

The isobaric heat capacity of DESs was determined experimentally. Table S10 shows, for all pure DESs used in this work, the thermal expansion coefficient, the isobaric heat capacity and the Flory parameters necessary for the application of the PFP theory. The results of our experiments compare the Cp of DES1–DES4. Quite noticeable differences are observed. Generally, the *Cp* values [J·mol^−1^ K^−1^] are arranged in order: DES 3 < DES 2 < DES 1 < DES 4 at set temperature points.

According to the PFP theory, for the separation of the values of excess molar volume into three contributions, the interactional parameter *χ*_12_ must be found. In the present study, it has been done by minimalization of the objective function, considering deviations in the prediction of the excess volume, defined as

(13) $$OF=\overset{n}{\underset{i=1}{\sum }}{\left(V_{exp}^{E}-V_{calc}^{E}\right)}^{2}$$

**Figure 3:**
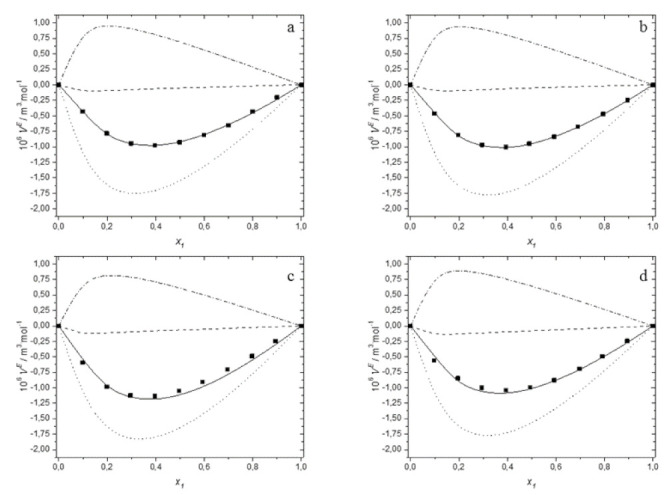
The dependence of the excess molar volume of aqueous solutions of DESs on molar fraction of deep eutectic solvent at 298.15 K: (**a**) for DES1; (**b**) for DES2; (**c**) for DES3; (**d**) for DES4; (■) experimental data; (**―**) calculated using the PFP model; (**………**) interactional contribution ($V_{int}^{E}$); (**– – –**), free volume contribution ($V_{fv}^{E}$); (**– ▪ –**), characteristic pressure contribution ($V_{P^{*}}^{E}$).

In calculations, the value of the interactional parameter *χ*_12_ was assumed to be independent on the composition of mixture. Figure 3 shows the composition dependence of calculated excess molar volume, together with the three contributions ($V_{int}^{E},V_{fv}^{E},V_{P^{*}}^{E}$), compared with the experimental *V^E^* data for each system studied at 298.15 K.

Moreover, Table 3 reports the adjusted values of interactional parameter *χ*_12_ and calculated three contributions to excess molar volume for the binary mixtures of deep eutectic solvents with water at all temperatures investigated and at *x*_1_ = 0.4 together with RMSD.

Study of the data presented in Table 3 as well as an analysis of Figure 3 reveals that the interactional contribution is always negative and it seems to be the most important to explain the values of the excess molar volume. It decides about the sign and magnitude of the *V^E^* due to its greater value compared to the other two contributions for all investigated systems at all temperatures. The free volume contribution, which is a measure of geometrical accommodation, is negative but its magnitude is much smaller than for the interactional contributions. Therefore, it can be said that the PFP model confirms the conclusions resulting from the dependence of excess molar volume on the temperature, postulating little significance of the packing effect for the systems studied. The third contribution is the result of differences in internal pressure and in the reduced volumes of the components. It is positive, its magnitude is smaller than the interactional contribution and decreases distinctly with temperature.

**Figure 4:**
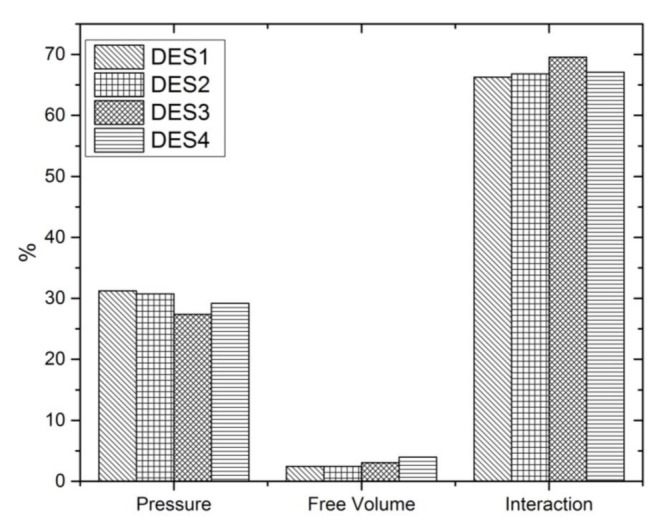
Percentages of the three contributions in excess molar volume of aqueous solutions of DESs for *x*_1_ = 0.4 at 298.15 K.

For deeper analysis of the obtained results, the percentage of the three contributions in excess molar volume was calculated. Figure 4 presents the obtained results at 298.15 K.

As can be seen, the interactional contribution and the characteristic pressure contribution determine the order of the excess molar volume observed for the studied systems, which is as follows: TBAB:MAE (DES 3) < TBAB:BAE (DES 4) < TBAC:AP (DES 2) ≈ TBAB:AP (DES 1). The free volume fraction has practically no effect on the excess molar volume, and its absolute value increases with the length of the alkyl chain in the amino alcohol.

Moreover, the results show that the anion of the salt in DES does not practically effect on the value of excess molar volume. As depicted in Figure 3 and Figure 4, almost identical interactional contribution, free volume contribution and characteristic pressure contribution are observed for aqueous solutions of TBAC:AP (DES 2) and TBAB:AP (DES 1).

Further analysis of Table 3 shows that the interactional contribution is mainly responsible for the increase of excess molar volume with increasing temperature. The decrease in its absolute value is greater than the decrease of the positive characteristic pressure contribution, and consequently the excess volumes of the studied systems increase with temperature. The percentage of free volume contribution increases with increasing temperature, but due to the low absolute values $V_{int}^{E}$, it has no influence on the excess molar volume or its dependence on temperature.

Summing up, it is evident from Figure 3 that the PFP theory predicts the experimental data satisfactorily. Thus, while the PFP theory does not take into account the strong interactions between components—such as electrostatic, hydrogen bonding, and complex formation—we can infer that the PFP model reproduces the main characteristics of the experimental data by using only one fitted parameter to describe excess molar volume.

#### 3.2.2. Excess of Thermal Expansion

As the temperature dependence of density was found to be second order polynomial, type: $\ln\left(\rho \right)=at^{2}+bt+c$, the isobaric thermal expansion coefficients at different temperatures were derived according to the equation

(14) $$\alpha_{p}=\frac{1}{V}{\left(\frac{\delta V}{\delta T}\right)}_{P}=-\frac{1}{\rho }{\left(\frac{\delta \rho }{\delta T}\right)}_{P}=-{\left(\frac{\delta ln\rho }{\delta T}\right)}_{P}=-\left(2a+b\right)$$

**Figure 5:**
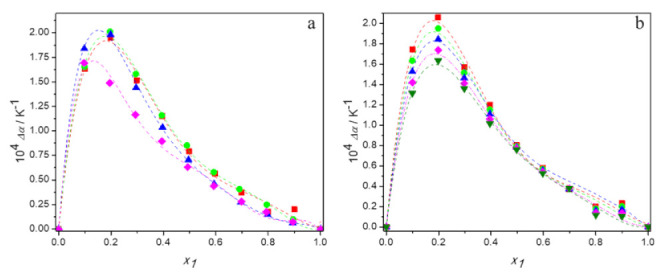
Dependence of the excess thermal expansion of aqueous solutions of DESs on molar fraction of deep eutectic solvent: (**a**) at 298.15 K for ■ DES1; ● DES2; ▲ DES3; ♦ DES4; (**b**) for DES1 at 293.15 K (■); 298.15 K (●); 303.15 K (▲); 308.15 K (♦); 313.15 K (▼); —, Equation (6).

Then, excess thermal expansion, Δ*α_p_*, was calculated using the equation

(15) $$\Delta \alpha_{p}=\alpha_{p}-\overset{n}{\underset{i=1}{\sum }}\Phi_{i}\alpha_{p,i}$$

where Φ*_i_* is the volume fraction of pure component *i*, defined as $\phi_{i}=x_{i}V_{i}/\sum_{i}x_{i}V_{i,}$. The values of *α_p_* and Δ*α_p_* are given in Tables S1–S6 in Supporting Material and variation of the excess thermal expansion with DES mole fraction, *x*_1_, is plotted in Figure 5.

It can be seen that the values of excess thermal expansion are positive in the entire composition range for all systems studied, regardless of temperature. Since positive Δ*α_p_* are typical for the systems containing molecules capable to self-associate, the obtained results confirm strong hydrogen bonds between water molecules or between molecules of deep eutectic solvents, the strength of which decreases with temperature, as indicated by a reduction in excess thermal expansion with temperature [53]. Moreover, the less positive values of excess thermal expansion obtained for the (TBAB:BAE (DES 4) + water) system indicate the weakest hydrogen bond interactions between molecules of this DES compared to the others.

#### 3.2.3. Partial Molar Volumes

The partial molar volumes of the studied DESs and water in their binary mixtures, ${\bar{V}}_{1}$ and ${\bar{V}}_{2}$, were calculated from the Equations (16) and (17), using the parameters of Redlich Kister equation (Tables S6–S9) and the molar volumes of the pure components, $V_{1}^{o}$ and $V_{2}^{o}$

(16) $${\bar{V}}_{1}=V_{1}^{o}+{\left(x_{1}-1\right)}^{2}\overset{j}{\underset{i=0}{\sum }}A_{i}{\left(2x_{1}-1\right)}^{i}+2x_{1}{\left(1-x_{1}\right)}^{2}\overset{j}{\underset{i=0}{\sum }}A_{i}i{\left(2x_{1}-1\right)}^{i-1}$$

(17) $${\bar{V}}_{2}=V_{2}^{o}+x_{1}{}^{2}\overset{j}{\underset{i=0}{\sum }}A_{i}{\left(2x_{1}-1\right)}^{i}-2x_{1}{}^{2}\left(1-x_{1}\right)\overset{j}{\underset{i=0}{\sum }}A_{i}i{\left(2x_{1}-1\right)}^{i-1}$$

**Figure 6:**
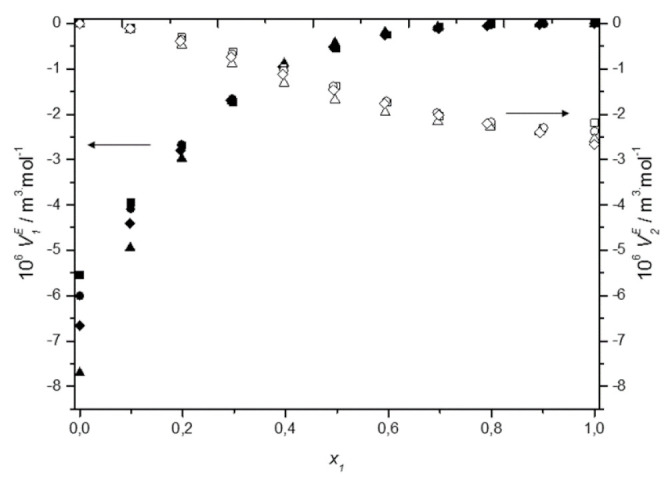
Dependence of the excess partial molar volume ${\bar{V}}_{1}^{E}$ of ■ DES1, ● DES2, ▲ DES3, ♦ DES4, and water ${\bar{V}}_{2}^{E}$ in □ DES1, ○ DES2, △ DES3, ◊ DES4 on molar fraction of deep eutectic solvent at 298.15 K.

The obtained values of the partial molar volumes together with the molar volumes of pure components are presented in Table S11 in Supplementary Material. As can be seen, at all studied temperatures, the molar volumes of the interacting compounds in the pure state were higher than their corresponding values in the mixture, indicating the reduction in volume upon adding a deep eutectic solvent to water. Figure 6 shows the excess partial molar volumes of the components at 298.15 K. These properties were calculated from definition as: ${\bar{V}}_{i}^{E}={\bar{V}}_{i}-V_{i}^{o}$ and their values are negative over the whole composition range. In general, the negative ${\bar{V}}_{1}^{E}$ and ${\bar{V}}_{2}^{E}$ values indicate the presence of significant solute–solvent interactions between unlike molecules, whereas the positive ${\bar{V}}_{1}^{E}$ and ${\bar{V}}_{2}^{E}$ values indicate the presence of solute–solute or solvent–solvent interactions between like molecules in the mixture [54]. In the present work, the negative excess partial molar volumes of the components indicate that the DES—water interactions are stronger than the DES—DES or the water–water interactions, what is consistent with the conclusions from excess molar volumes.

Since the partial molar properties at infinite dilution provide useful information about the interactions between components of a mixture that are independent on composition, their values for DES and water were calculated.

The partial molar volumes at infinite dilution of DES were obtained by setting $x_{1}=0$ in Equation (18) as

(18) $${\bar{V}}_{1}^{\infty }=V_{1}^{o}+\overset{j}{\underset{i=0}{\sum }}A_{i}{\left(-1\right)}^{i}$$

Similarly, setting $x_{1}=1$ in Equation (19) allowed to estimate the partial molar volumes at infinite dilution of water

(19) $${\bar{V}}_{2}^{\infty }=V_{2}^{o}+\overset{j}{\underset{i=0}{\sum }}A_{i}$$

Table 4 presents the obtained values of partial molar volumes at infinite dilutions of DES and water in their binary systems.

As can be seen, both the partial molar volumes and the excess partial molar volumes at infinite dilution increase with the increasing temperature. Such results seem to indicate that the weakening of hydrogen bond interactions between DES and water molecules with increase in temperature is the most important factor controlling the properties of the systems and it dominates over the packing effect. Moreover, the excess partial molar volumes at infinite dilution of DES change in the order TBAB:MAE (DES 3) < TBAB:BAE (DES 4) < TBAC:AP (DES 2) < TBAB:AP (DES 1) confirming the conclusions obtained on the basis of excess molar volumes. The dependence of excess partial molar volumes at infinite dilution of water on the DES: TBAB:BAE (DES 4) ≤ TBAB:MAE (DES 3) < TBAC:AP (DES 2) < TBAB:AP (DES 1) is similar and only the reordering of DES4 and DES3 takes place. However, taking into an account the uncertainty of partial molar volume at infinite dilution, it can be said, that the ${\bar{V}}_{2}^{E\infty }$ for systems (DES4+water) and (DES3+water) are practically equal.

### 3.3. Excess Isentropic Compressibility

The isentropic compressibilities of aqueous solutions of DESs were estimated using experimental values of densities and sound velocities by the Laplace equation

(20) $$\kappa_{S}=-\frac{1}{V_{m}}{\left(\frac{\partial V_{m}}{\partial P}\right)}_{S}=\frac{1}{u^{2}\cdot \rho }$$

providing the link between thermodynamics and acoustics.

Then, according to the approach developed by Benson et al. [55], the excess isentropic compressibility was calculated as

(21) $$\kappa_{S}^{E}=\kappa_{S}-\underset{i}{\sum }\Phi_{i}\kappa_{S,i}-T\underset{i}{\sum }\Phi_{i}V_{i}\alpha_{p,i}^{2}/C_{p,i}+T\underset{i}{\sum }x_{i}V_{i}{\left(\underset{i}{\sum }\Phi_{i}\alpha_{p,i}\right)}^{2}/\underset{i}{\sum }x_{i}C_{p,i}$$

**Figure 7:**
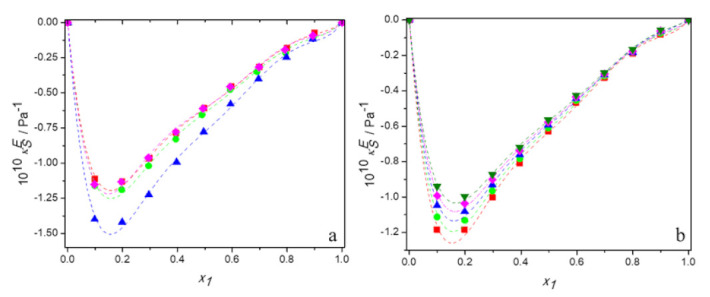
Dependence of the excess isentropic compressibility of aqueous solutions of DESs on molar fraction of deep eutectic solvent: (**a**) at 298.15 K for ■ DES1; ● DES2; ▲ DES3; ♦ DES4; (**b**) for DES1 at 298.15 K (■); 298.15 K (●); 303.15 K (▲); 308.15 K (♦); 313.15 K (▼); —, Equation (6).

Tables S1–S4 in Supplementary Material present the experimental values of sound velocity and the calculated values of excess isentropic compressibility for aqueous solutions of DESs made of tetrabutylammonium bromide and 3-amino-1-propanol or 2-(methylamino)ethanol or 2-(butylamino)ethanol and for the aqueous solutions of DES built of tetrabutylammonium chloride and 3-amino-1-propanol over the entire range of compositions at temperatures ranging from 293.15 K to 313.15 K. Figure 7a shows the plots of $\kappa_{S}^{E}$ against mole fraction of DES for the all studied mixtures at 298.15 K and Figure 7b depicts the temperature dependence of excess isentropic compressibility for the system (TBAB:AP +water) as an example. It is evident that the curves are remarkably asymmetric, with their minima shifted towards a rich mole fraction of water, even more than the excess molar volume curves are.

In these figures, it can be also seen that the values of $\kappa_{S}^{E}$ are negative for all systems over the entire range of the mole fraction as well as the temperature range. This indicates that the mixtures might be less compressible than the corresponding ideal mixtures due to a closer approach and stronger interactions between the unlike molecules of the mixtures. The negative values of excess isentropic compressibility for the binary systems follow the order: TBAB:MAE (DES 3) < TBAB:BAE (DES 4) ≈ TBAC:AP (DES 2) ≈ TBAB:AP (DES 1). Thus, the behavior of the excess isentropic compressibility seems to be consistent with the obtained values of the excess molar volume, which suggests that the interactions and the packing effect dominate in aqueous solutions of TBAB:MAE and do not practically depend on the anion of the salt in DES.

Moreover, as can be seen in Figure 7b, the values of $\kappa_{S}^{E}$ become less negative with increasing temperature for all systems at a fixed composition. It is due to the reduction of interactions between unlike molecules, what has already been suggested by volumetric properties. Indeed, the increase in temperature also increases the thermal motion of the molecules and enlargement of interstices. However, the decrease in hydrogen bonding is greater and, in the result, the excess molar compressibility of all aqueous solutions of DES decreases with increasing temperature.

### 3.4. Deviations in Refractive Index

From refractive indices the deviations in refractive index, Δ*n_D_*, were calculated using the equation

(22) $$\Delta n_{D}=n_{D}-\overset{n}{\underset{i=1}{\sum }}\Phi_{i}n_{Di}$$

where *n_D_* and *n_Di_* are the refractive index of a mixture and a pure component *i*, respectively and Φ*_i_* denotes the volume fraction of a pure component *i*.

**Figure 8:**
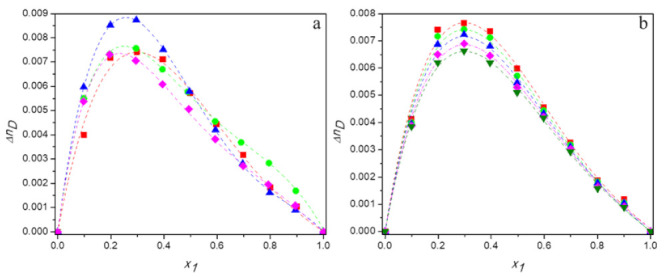
Dependence of the deviations in refractive of aqueous solutions of DESs on molar fraction of deep eutectic solvent: (**a**) 298. 15 K for ■ DES1; ● DES2; ▲ DES3; ♦ DES4; (**b**) for DES1 at 298.15 K (■); 298.15 K (●); 303.15 K (▲); 308.15 K (♦); 313.15 K (▼); —, Equation (6).

Tables S1–S4 in Supplementary Material present the experimental values of refractive index and calculated values of the deviations in refractive index for aqueous solutions of DESs over the entire range of compositions at temperatures ranging from 293.15 K to 313.15 K. Figure 8a shows the plots of $\Delta n_{D}$ against mole fraction of DES for the all studied mixtures at 298.15 K and Figure 8b depicts the temperature dependence of deviations in refractive index for the system (TBAB:AP +water) as an example.

As can be seen from Figure 8, the values of deviations in refractive index are positive over the whole composition range of binary mixtures and their dependences on mole fraction of DES are asymmetrical.

It is known that deviations of refractive index are negatively correlated with excess molar volumes [56]. If excess molar volume is negative, then there will be less free volume available than in an ideal mixture and the photons will interact more strongly with the components of the solution. As a result, light will travel with a weaker velocity in the mixture and its refractive index will be higher than in an ideal solution. Thus, positive deviations of refractive index will be observed.

This phenomenon occurs in all systems investigated in the present study and it is the strongest for TBAB:MAE (DES 3). Therefore, the obtained deviations of refractive index confirm the conclusion regarding the strongest interactions between this deep eutectic solvent and water molecules compared to other DESs studied. Moreover, as the values Δ*n_D_* increase with decreasing temperature, they indicate an increase in the number of hydrogen bonds at lower temperatures, which corresponds with the results of densitometric and acoustic research.

### 3.5. Deviations in Viscosity and Excess Gibbs Energy of Activation for Viscous Flow

Based on the viscosities of the mixtures, the viscosity deviations Δ*η* were obtained according to the equation

(23) $$\Delta \eta =\eta -\exp(x_{1}ln\eta_{1}+x_{2}ln\eta_{2})$$

which uses the viscosity of the ideal mixture as suggested by Arrhenius.

The excess Gibbs energy of activation for viscous flow Δ*G^E^*, was calculated using the equation

(24) $$\Delta G^{E}=RT\left[\ln\left(\eta V_{m}\right)-(x_{1}\ln\left(\eta_{1}V_{1}\right)+x_{2}\ln\left(\eta_{2}V_{2}\right)\right]$$

where *R* is the gas constant and *T* is the absolute temperature. The symbols *η*_1_, *η*_2_, *V*_1_, and *V*_2_ represent viscosity of DES, viscosity of water, molar volume of DES, and molar volume of water, respectively.

**Figure 9:**
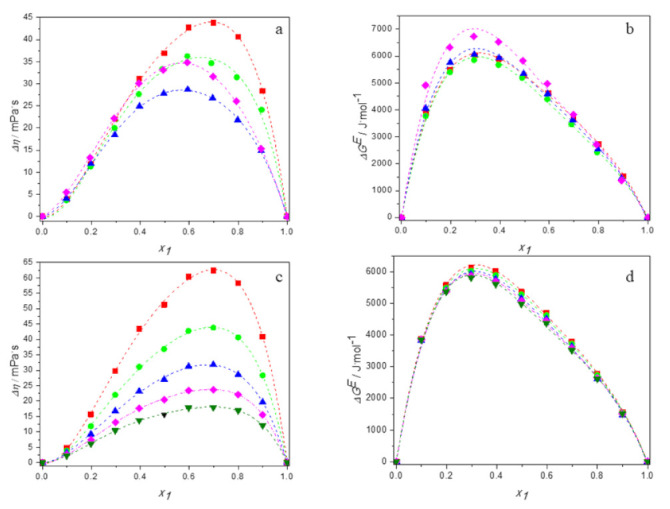
Dependence of the deviations in viscosity and the excess Gibbs energy of activation of aqueous solutions of DESs on molar fraction of deep eutectic solvent: (**a**,**b**) at 298.15 K for ■ DES1; ● DES2; ▲ DES3; ♦ DES4; (**c**,**d**) for DES1 at 298.15 K (■); 298.15 K (●); 303.15 K (▲); 308.15 K (♦); 313.15 K (▼); —, Equation (6).

Tables S1–S4 in Supplementary Material present the experimental values of viscosity, calculated values of the viscosity deviations and the excess Gibbs energy of activation for viscous flow for aqueous solutions of DESs over the entire range of compositions at temperatures ranging from 293.15 K to 313.15 K. Figure 9a,b show the plots of Δη and $\Delta G^{E}$ against mole fraction of DES for the all studied mixtures at 298.15 K. Figure 9c,d depict the temperature dependence of viscosity deviations and the temperature dependence of the excess Gibbs energy of activation for viscous flow for the system (TBAB:BAE +water) as an example.

It is clearly visible that the viscosity deviations of all mixtures are positive in the whole range of composition. It is known that the viscosity of a mixture is related to the liquid structure [57]. Therefore, the viscosity deviation depends on molecular interactions as well as on the size and shape of the molecules forming the solution. The positive viscosity deviations are observed in mixtures with strong specific interactions like hydrogen bonding interactions, whereas the interstitial accommodation of one component with the other within the mixture leads to negative Δ*η* values [58]. For our DES systems, the predominant effect is the hydrogen bonding, that leads to positive Δ*η* values.

The order of viscosity deviations is the same as for the viscosity of the studied systems and is as follows: TBAB:AP (DES 1) > TBAC:AP (DES 2) > TBAB:BAE (DES 4) > TBAB:MAE (DES 3). It is different from those obtained for excess molar volume or excess compressibility. Thus, it can concluded that the values of viscosity deviations are determined not only by the interactions between unlike molecules, but also by other effects as shape of the molecules.

Estimation of the results obtained and presented in Figure 9 confirm the temperature dependence of the viscosity deviations in the studied systems because the values of Δ*η* become less positive with increasing temperature. This is due to the weakening of the interactions between the molecules present in the solution, which seem to dominate over the penetration phenomenon that obviously increases with temperature.

The positive values of excess Gibbs energy of activation presented in Figure 9a,b once again approve the dominance of specific interactions—i.e., hydrogen bonding between DES and water molecules occurring in the studied systems—which become weaker as temperature increases [58].

### 3.6. Correlations of Excess Properties

Similarly, as in a case of excess molar volumes, in order to correlate the calculated excess thermal expansions, excess isentropic compressibilities, deviations in refractive index, deviations in viscosity, and excess Gibbs energy of activation for viscous flow with the composition, the Redlich–Kister polynomial equation was applied [34]

(25) $$Y^{E}=x_{1}x_{2}\overset{2}{\underset{i=0}{\sum }}A_{i}{\left(x_{1}-x_{2}\right)}^{i}$$

where the *A_i_* values are adjustable parameters. They were determined using the least squares method and their values are listed in Tables S6–S9 along with root mean square deviations of fit (RMSD). In order to obtain RMSD close to the experimental uncertainty, a different degree of the polynomial equation was chosen depending on the property and DES. For almost all systems, excess molar volumes, deviations in refractive index, and excess Gibbs energy of activation for viscous flow with composition were correlated using free-parameter Redlich–Kister polynomial equation. For viscosity deviations and excess isentropic compressibilities, a better fit was obtained for four-parameter and for five-parameter Redlich–Kister polynomial equation, respectively. In case of excess thermal expansions four-parameter for DES1 and DES 2 and five-parameter Redlich–Kister polynomial for DES3 and DES5 were chosen.

In Figure 2, Figure 5, Figure 7, Figure 8 and Figure 9, the dashed lines represent the correlated values according to the Redlich–Kister equation. As it can be seen, the calculated values agree very well with the experimental data. Thus, the Redlich–Kister equation perfectly represents the data over the experimental temperature range for the novel DES + water binary systems studied in this work.

## 4. Conclusions

The presented novel DESs built of tetrabutylammonium chloride and 3-amino-1-propanol or tetrabutylammonium bromide and 3-amino-1-propanol or 2-(methylamino)ethanol or 2-(butylamino)ethanol were found to be attractive in their properties, mostly for further evaluation and optimization during development of inexpensive eco-solvents or other valuable material. Most important physicochemical properties have been demonstrated in details such as density, speed of sound, refractive index, and viscosity which were measured for their aqueous solutions over the entire range of compositions at atmospheric pressure and T = (293.15 − 313.15). The chosen Jouyban–Acree model was successfully used to correlate the experimental physical properties with respect to the concentration, and the results showed that this mathematical equation is an accurate correlation for the prediction of aqueous DES properties.

Excess molar volumes, excess isentropic compressibilities, deviations in viscosity, and deviations in refractive indices were calculated to study nonideal behavior of binary mixtures and they were correlated by the Redlich–Kister equation with temperature-dependent parameters. Excess molar volumes and excess compressiblities were negative and deviations in viscosity and deviations in refractive index were positive over the entire range of composition and temperature, suggesting strong intermolecular interactions among unlike molecules. Moreover, the temperature dependences of the excess molar volumes and compressibilities indicate that, in the studied systems, hydrogen bonding prevails over the packing effect (non-specific interactions).

The dominance of specific interactions in the aqueous solutions of the DESs also was confirmed by the Prigogine–Flory–Patterson (PFP) theory, which was applied to excess molar volumes.

The calculated negative values of the excess partial molar volumes of DESs and water demonstrated sufficient DES—water interactions which are stronger than the DES—DES or the water–water ones will probably facilitate the efficient utilization of DES.

In terms of the suitability of the water mixtures of the studied DES for the effective separation of carbon dioxide from gas streams at relatively low pressure, the obtained values of the excess properties allow us to assume that the best absorbent would be TBAB: AP, and the worst of TBAB:MAE.

## Figures and Tables

**Table 1:** Provenance, mass fraction purity, and chemical structures of the compounds studied.

Chemical Name	Source	CAS Number	Molecular Weight M/(g·mol^−1^)	Mass Fraction Purity	Chemical Structure
3-amino-1-propanol (AP)	Sigma Aldrich	156-87-6	75.11	0.99 ^a^	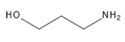
2-(methylamino)-ethanol (MEA)	Sigma Aldrich	109-83-1	75.11	≥98 ^a^	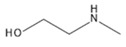
2-(butylamino)-ethanol (BEA)	Sigma Aldrich	111-75-1	117.19	≥98 ^a^	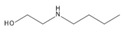
tetrabutylammonium bromide (TBAB)	Sigma Aldrich	1643-19-2	322.37	≥0.99 ^a^	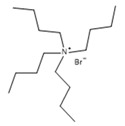
tetrabutylammonium chloride (TBAC)	Sigma Aldrich	1112-67-0	277.92	≥0.98 ^b^	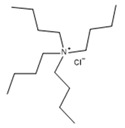

^a^ As stated by supplier. ^b^ After crystalization, determined by potentiometric titration.

**Table 2:** Abbreviations of chemicals and DESs along with their molar mass, molar ratio, mass fraction, and water content.

Symbol	HBA	HBD	Molar Ratio	M_DES_/ (g·mol^−1^)	Mass Fraction of HBA ^a^	Water Content ^b^
DES1	TBAB	AP	1:6	110.449	0.4172	0.00059
DES2	TBAC	AP	1:6	104.091	0.3815	0.00121
DES3	TBAB	MAE	1:6	110.433	0.4170	0.00118
DES4	TBAB	BAE	1:6	146.519	0.3145	0.00159

^a^ The standard uncertainty of DES mass fraction composition is 0.0001. ^b^ Water content of DESs in mass fraction determined by Karl Fisher titration with the standard uncertainty ±0.0001.

**Table 3:** Calculated values of the interactional parameter χ12, root mean square deviation of fit RMSD, and the three contributions ($V_{int}^{E},V_{fv}^{E},V_{P^{*}}^{E}$) from the PFP theory to the excess molar volumes for the binary mixtures of deep eutectic solvents with water at x1 = 0.4 and T = (293.15 − 313.15) K.

*T*/K	293.15	298.15	303.15	308.15	313.15
	DES_1_ (1) + water (2)				
10^−6^ χ_12_/J·m^−3^	−393.3	−354.2	−317.6	−283.2	−252.1
10^6^ $V_{int}^{E}$/m^3^·mol^−1^	−1.932	−1.726	−1.534	−1.359	−1.205
10^6^ $V_{P^{*}}^{E}$/m^3^·mol^−1^	0.979	0.814	0.661	0.522	0.398
10^6^ $V_{fv}^{E}$/m^3^·mol^−1^	−0.062	−0.064	−0.066	−0.059	−0.053
RMSD	0.018	0.016	0.022	0.033	0.046
	DES_2_ (1) + water (2)				
10^−6^ χ_12_/J·m^−3^	−397.7	−358.5	−321.7	−287.2	−256.0
10^6^ $V_{int}^{E}$/m^3^·mol^−1^	−1.960	−1.752	−1.559	−1.384	−1.230
10^6^ $V_{P^{*}}^{E}$/m^3^·mol^−1^	0.964	0.806	0.652	0.513	0.390
10^6^ $V_{fv}^{E}$/m^3^·mol^−1^	−0.062	−0.065	−0.064	−0.060	−0.053
RMSD	0.020	0.006	0.008	0.020	0.033
	DES_3_ (1) + water (2)				
10^−6^ χ_12_/J·m^−3^	−324.1	−294.1	−266.0	−241.2	−218.9
10^6^ $V_{int}^{E}$/m^3^·mol^−1^	−2.031	−1.818	−1.624	−1.454	−1.305
10^6^ $V_{P^{*}}^{E}$/m^3^·mol^−1^	0.888	0.716	0.559	0.421	0.301
10^6^ $V_{fv}^{E}$/m^3^·mol^−1^	−0.078	−0.081	−0.079	−0.074	−0.067
RMSD	0.080	0.068	0.054	0.040	0.029
	DES_4_ (1) + water (2)				
10^−6^ χ_12_/J·m^−3^	−245.4	−217.4	−190.9	−166.9	−145.5
10^6^ $V_{int}^{E}$/m^3^·mol^−1^	−1.989	−1.750	−1.529	−1.332	−1.158
10^6^ $V_{P^{*}}^{E}$/m^3^·mol^−1^	0.946	0.761	0.591	0.438	0.302
10^6^ $V_{fv}^{E}$/m^3^·mol^−1^	−0.091	−0.096	−0.097	−0.094	−0.087
RMSD	0.062	0.045	0.031	0.027	0.033

**Table 4:** Partial molar volumes at infinite dilution of DES and water in their binary mixtures at *T* = (293.15 to 313.15) K and at atmospheric pressure (0.1 MPa).

*T*/K	$10^{6}\;{\bar{V}}_{1}^{\infty }/\;m^{3}\cdot {\mathrm{mol}}^{-1}$	$10^{6}\;{\bar{V}}_{1}^{E\infty }/\;m^{3}\cdot {\mathrm{mol}}^{-1}$	$10^{6}\;{\bar{V}}_{2}^{\infty }/\;m^{3}\cdot {\mathrm{mol}}^{-1}$	$10^{6}\;{\bar{V}}_{2}^{E\infty }/\;m^{3}\cdot {\mathrm{mol}}^{-1}$	$10^{6}\;{\bar{V}}_{1}^{\infty }/\;m^{3}\cdot {\mathrm{mol}}^{-1}$	$10^{6}\;{\bar{V}}_{1}^{E\infty }/\;m^{3}\cdot {\mathrm{mol}}^{-1}$	$10^{6}\;{\bar{V}}_{2}^{\infty }/\;m^{3}\cdot {\mathrm{mol}}^{-1}$	$10^{6}\;{\bar{V}}_{2}^{E\infty }/\;m^{3}\cdot {\mathrm{mol}}^{-1}$
	DES 1 + water				DES 2 + water			
293.15	102.91	−5.83	15.94	−2.11	101.10	−6.34	15.58	−2.47
298.15	103.60	−5.54	15.88	−2.19	101.82	−6.01	15.69	−2.38
303.15	104.32	−5.22	16.13	−1.96	102.51	−5.71	15.79	−2.30
308.15	105.00	−4.95	16.24	−1.88	103.18	−5.44	15.89	−2.24
313.15	105.65	−4.70	16.31	−1.85	103.84	−5.19	15.98	−2.17
	**DES 3 + water**				**DES 4 + water**			
293.15	104.00	−8.04	15.45	−2.6	149.34	−6.98	15.29	−2.75
298.15	104.75	−7.70	15.52	−2.54	150.27	−6.66	15.40	−2.67
303.15	105.47	−7.39	15.60	−2.49	151.17	−6.36	15.50	−2.60
308.15	106.18	−7.10	15.69	−2.43	152.06	−6.09	15.59	−2.53
313.15	106.88	−6.83	15.77	−2.39	152.94	−5.83	15.68	−2.47

## Data Availability

The data presented in this study are available on request from the corresponding author. The data are not publicly available due to the lack of requirements of Gdansk University of Technology and Medical University of Gdansk.

## Supplementary Materials

The following supporting information can be found online: Table S1: Densities *ρ*, excess molar volumes *V^E^*, isobaric thermal expansion coefficients *α_p_*, excess thermal expansion Δ*α_p_*, speeds of sound u, excess isentropic compressibilities $\kappa_{S}^{E}$, viscosities *η*, viscosity deviations Δ*η*, excess Gibbs free energy of activation of viscous flow Δ*G^E^*, refractive indices *n_D_*, refractive index deviations Δ*n_D_* as functions of mole fraction, *x*_1_ of DES for TBAB:AP (DES1) + water mixtures at the temperatures (293.15 to 303.15) K and atmospheric pressure; Table S2: Densities *ρ*, excess molar volumes *V^E^*, isobaric thermal expansion coefficients *α_p_*, excess thermal expansion Δ*α_p_*, speeds of sound u, excess isentropic compressibilities $\kappa_{S}^{E}$, viscosities *η*, viscosity deviations Δ*η*, excess Gibbs free energy of activation of viscous flow Δ*G^E^*, refractive indices *n_D_*, refractive index deviations Δ*n_D_* as functions of mole fraction, *x*_1_ of DES for TBAC:AP (DES2) + water mixtures at the temperatures (293.15 to 303.15) K and atmospheric pressure; Table S3:Densities *ρ*, excess molar volumes *V^E^*, isobaric thermal expansion coefficients *α_p_*, excess thermal expansion Δ*α_p_*, speed of sounds u, excess isentropic compressibilities $\kappa_{S}^{E}$, viscosities *η*, viscosity deviations Δ*η*, excess Gibbs free energy of activation of viscous flow Δ*G^E^*, refractive indices *n_D_*, refractive index deviations Δ*n_D_* as functions of mole fraction, *x*_1_ of DES for TBAB:MAE (DES3) + water mixtures at the temperatures (293.15 to 303.15) K and atmospheric pressure; Table S4: Densities *ρ*, excess molar volumes *V^E^*, isobaric thermal expansion coefficients *α_p_*, excess thermal expansion Δ*α_p_*, speed of sound u, excess isentropic compressibilities $\kappa_{S}^{E}$, viscosities *η*, viscosity deviations Δ*η*, excess Gibbs free energy of activation of viscous flow Δ*G^E^*, refractive indices *n_D_*, refractive index deviations Δ*n_D_* as functions of mole fraction, *x*_1_ of DES for TBAB:BAE (DES4) + water mixtures at the temperatures (293.15 to 303.15) K and atmospheric pressure; Table S5: Parameters of the JAM equation, together with RMSD and ARD% for density, speed of sound, viscosity and refractive index of DES (1) + water (2) systems at different temperatures; Table S6: Parameters *A_i_* of Equation (6) and the corresponding RSMD f for TBAB:AP (DES1) + water mixtures at the temperatures (293.15 to 303.15) K and atmospheric pressure; Table S7. Parameters *A_i_* of Equation (6) and the corresponding RSMD for TBAC:AP (DES2) + water mixtures at the temperatures (293.15 to 303.15) K and atmospheric pressure; Table S8: Parameters *A_i_* of Equation (6) and the corresponding RSMD for TBAB:MAE (DES3) + water mixtures at the temperatures (293.15 to 303.15) K and atmospheric pressure; Table S9: Parameters *A_i_* of Equation (6) and the corresponding RSMD for TBAB:BAE (DES4) + water mixtures at the temperatures (293.15 to 303.15) K and atmospheric pressure; Table S10: Isobaric thermal expansion coefficient (*α_p_*), isochoric molar heat capacity (C_P_), Flory theory parameters: characteristic volume (V*), reduce volume (Ṽ), characteristic pressure (P*), and ratio of molecular surface to volume ratio (S1/S2) of DES to water; Table S11: Partial molar volumes of DESs and water in their binary mixtures at T = (293.15 to 313.15) K and at atmospheric pressure (0.1 MPa).
