# Supplementary material for: Novel Binary Mixtures of Alkanolamine Based Deep Eutectic Solvents with Water—Thermodynamic Calculation and Correlation of Crucial Physicochemical Properties
Source: Molecules. 2022 Jan 25;27(3):788. doi: 10.3390/molecules27030788 (PMC8840229; doi:10.3390/molecules27030788)
Supplement: Supplementary file 1 [file molecules-27-00788-s001.zip › molecules-1554526-supplementary.pdf]

# Novel binary mixtures of alkanolamine based deep eutectic solvents with water - thermodynamic calculation and correlation of crucial physicochemical properties

Bartosz Nowosielski[a], Marzena Jamrógiewicz[b], Justyna Łuczak[c], Dorota Warmińska[a*]

[a] Department of Physical Chemistry, Faculty of Chemistry, Gdańsk University of Technology, ul. Narutowicza 11/12, 80-233 Gdańsk, POLAND

[b] Department of Physical Chemistry, Faculty of Pharmacy, Medical University of Gdańsk, Al. Gen. Hallera 107, 80-416 Gdańsk, POLAND

[c] Department of Process Engineering and Chemical Technology, Faculty of Chemistry, Gdańsk University of Technology, ul. Narutowicza 11/12, 80-233 Gdańsk, POLAND

[*]Corresponding author: Tel.:+48583471410; fax:+48583472694,

E-mail address: dorwarmi@ pg.edu.pl

Table S1. Densities $\rho$, excess molar volumes $V^E$, isobaric thermal expansion coefficients $\alpha_p$ , excess thermal expansion $\Delta\alpha_p$, speeds of sound $u$, excess isentropic compressibilities $k_S^E$, viscosities $\eta$, viscosity deviations $\Delta\eta$, excess Gibbs free energy of activation of viscous flow $\Delta G^{*E}$, refractive indices $n_D$, refractive index deviations $\Delta n_D$ as functions of mole fraction, $x_1$ of DES for TBAB:AP (DES1) + water mixtures at the temperatures (293.15 to 303.15) K and atmospheric pressure. [a]

| $x_1$ | $\rho$ / kg·m$^{-3}$ | $10^6\ V_m^E$/ m$^3$·mol$^{-1}$ | $10^4\ \alpha_p$ / $K^{-1}$ | $10^4\ \Delta\alpha_p$ / $K^{-1}$ | $u$ / m·s$^{-1}$ | $10^{10}\ k_S^E$ / Pa$^{-1}$ | $\eta$ / mPa·s | $\Delta\eta$ / mPa·s | $\Delta G^{*E}$ / J·mol$^{-1}$ | $n_D$ | $\Delta n_D$ |
|---|---|---|---|---|---|---|---|---|---|---|---|
| 293.15 K | | | | | | | | | | | |
| 0.0000 | 998.20 | 0.000 | 2.119 | 0.000 | 1482.52 | 0.000 | 1.05 | 0.000 | 0.00 | 1.3340 | 0.00000 |
| 0.0994 | 1022.32 | -0.453 | 5.944 | 1.742 | 1767.38 | -1.185 | 6.47 | 4.843 | 3874.91 | 1.3950 | 0.00413 |
| 0.1981 | 1032.09 | -0.817 | 7.296 | 2.057 | 1811.29 | -1.185 | 18.21 | 15.695 | 5585.67 | 1.4266 | 0.00742 |
| 0.2979 | 1033.23 | -0.979 | 7.437 | 1.569 | 1796.62 | -1.002 | 33.67 | 29.766 | 6123.90 | 1.4440 | 0.00764 |
| 0.3961 | 1031.40 | -1.006 | 7.477 | 1.196 | 1772.86 | -0.810 | 49.46 | 43.439 | 6022.89 | 1.4550 | 0.00736 |
| 0.4983 | 1028.66 | -0.950 | 7.390 | 0.802 | 1748.67 | -0.627 | 60.74 | 51.292 | 5373.26 | 1.4620 | 0.00599 |
| 0.5987 | 1025.68 | -0.827 | 7.393 | 0.581 | 1727.08 | -0.467 | 75.12 | 60.412 | 4709.86 | 1.4667 | 0.00456 |
| 0.6999 | 1022.93 | -0.668 | 7.356 | 0.368 | 1707.97 | -0.325 | 85.40 | 62.416 | 3790.44 | 1.4702 | 0.00326 |
| 0.8000 | 1020.02 | -0.445 | 7.325 | 0.198 | 1688.58 | -0.187 | 93.99 | 58.259 | 2775.90 | 1.4726 | 0.00187 |
| 0.9000 | 1017.55 | -0.211 | 7.473 | 0.233 | 1675.64 | -0.083 | 96.45 | 40.920 | 1565.90 | 1.4750 | 0.00118 |
| 1.0000 | 1015.71 | 0.000 | 7.334 | 0.000 | 1666.29 | 0.000 | 86.30 | 0.000 | 0.00 | 1.4764 | 0.00000 |
| *298.15* K | | | | | | | | | | | |
| 0.0000 | 997.05 | 0.000 | 2.565 | 0.000 | 1496.8 | 0.000 | 0.90 | 0.000 | 0.00 | 1.3331 | 0.00000 |
| 0.0994 | 1019.24 | -0.432 | 6.110 | 1.634 | 1757.08 | -1.113 | 5.25 | 3.879 | 3852.17 | 1.3937 | 0.00400 |
| 0.1981 | 1028.31 | -0.784 | 7.376 | 1.950 | 1796.54 | -1.131 | 13.92 | 11.837 | 5487.88 | 1.4250 | 0.00717 |
| 0.2979 | 1029.38 | -0.947 | 7.517 | 1.515 | 1781.00 | -0.965 | 25.20 | 22.023 | 6025.66 | 1.4423 | 0.00742 |
| 0.3961 | 1027.54 | -0.978 | 7.530 | 1.150 | 1756.80 | -0.783 | 35.95 | 31.134 | 5892.31 | 1.4532 | 0.00712 |
| 0.4983 | 1024.84 | -0.928 | 7.451 | 0.791 | 1732.43 | -0.609 | 44.31 | 36.886 | 5284.13 | 1.4601 | 0.00572 |
| 0.5987 | 1021.89 | -0.809 | 7.432 | 0.567 | 1710.67 | -0.454 | 54.10 | 42.745 | 4620.34 | 1.4649 | 0.00443 |
| 0.6999 | 1019.16 | -0.655 | 7.397 | 0.371 | 1691.51 | -0.317 | 61.29 | 43.856 | 3719.78 | 1.4684 | 0.00318 |
| 0.8000 | 1016.25 | -0.434 | 7.331 | 0.178 | 1671.67 | -0.181 | 67.26 | 40.626 | 2723.50 | 1.4708 | 0.00182 |
| 0.9000 | 1013.78 | -0.203 | 7.457 | 0.200 | 1657.12 | -0.072 | 69.02 | 28.345 | 1535.51 | 1.4731 | 0.00106 |
| 1.0000 | 1011.99 | 0.000 | 7.343 | 0.000 | 1649.50 | 0.000 | 62.12 | 0.000 | 0.00 | 1.4746 | 0.00000 |
| *303.15* K | | | | | | | | | | | |
| 0.0000 | 995.65 | 0.000 | 3.010 | 0.000 | 1509.53 | 0.000 | 0.79 | 0.000 | 0.00 | 1.3322 | 0.00000 |
| 0.0994 | 1016.09 | -0.412 | 6.275 | 1.526 | 1746.66 | -1.049 | 4.39 | 3.204 | 3835.67 | 1.3925 | 0.00394 |

| | | | | | | | | | | | |
|---|---|---|---|---|---|---|---|---|---|---|---|
| 0.1981 | 1024.50 | -0.752 | 7.456 | 1.844 | 1781.64 | -1.082 | 11.06 | 9.285 | 5408.30 | 1.4234 | 0.00687 |
| 0.2979 | 1025.50 | -0.918 | 7.597 | 1.463 | 1765.20 | -0.931 | 19.47 | 16.803 | 5921.86 | 1.4407 | 0.00723 |
| 0.3961 | 1023.66 | -0.951 | 7.583 | 1.105 | 1740.68 | -0.760 | 27.16 | 23.176 | 5766.31 | 1.4514 | 0.00680 |
| 0.4983 | 1021.02 | -0.906 | 7.513 | 0.781 | 1715.83 | -0.591 | 33.07 | 27.023 | 5155.10 | 1.4583 | 0.00546 |
| 0.5987 | 1018.09 | -0.792 | 7.471 | 0.553 | 1694.24 | -0.443 | 40.39 | 31.278 | 4518.31 | 1.4632 | 0.00433 |
| 0.6999 | 1015.39 | -0.642 | 7.438 | 0.374 | 1674.98 | -0.309 | 45.65 | 31.870 | 3634.30 | 1.4667 | 0.00310 |
| 0.8000 | 1012.58 | -0.433 | 7.336 | 0.157 | 1656.35 | -0.182 | 49.32 | 28.582 | 2618.91 | 1.4691 | 0.00178 |
| 0.9000 | 1009.99 | -0.193 | 7.441 | 0.168 | 1639.39 | -0.065 | 50.84 | 19.640 | 1459.93 | 1.4714 | 0.00104 |
| 1.0000 | 1008.28 | 0.000 | 7.351 | 0.000 | 1632.76 | 0.000 | 46.94 | 0.000 | 0.00 | 1.4729 | 0.00000 |
| *308.15* K | | | | | | | | | | | |
| 0.0000 | 994.03 | 0.000 | 3.455 | 0.000 | 1520.29 | 0.000 | 0.69 | 0.000 | 0.00 | 1.3314 | 0.00000 |
| 0.0994 | 1012.87 | -0.393 | 6.441 | 1.420 | 1736.11 | -0.992 | 3.72 | 2.698 | 3859.25 | 1.3913 | 0.00388 |
| 0.1981 | 1020.68 | -0.723 | 7.536 | 1.738 | 1766.28 | -1.038 | 8.96 | 7.451 | 5377.72 | 1.4217 | 0.00650 |
| 0.2979 | 1021.59 | -0.888 | 7.677 | 1.410 | 1749.37 | -0.901 | 15.33 | 13.092 | 5860.90 | 1.4389 | 0.00689 |
| 0.3961 | 1019.77 | -0.925 | 7.636 | 1.060 | 1724.41 | -0.739 | 21.05 | 17.752 | 5695.79 | 1.4495 | 0.00646 |
| 0.4983 | 1017.18 | -0.885 | 7.575 | 0.771 | 1699.51 | -0.578 | 25.34 | 20.402 | 5080.02 | 1.4565 | 0.00528 |
| 0.5987 | 1014.28 | -0.775 | 7.511 | 0.540 | 1677.87 | -0.434 | 30.69 | 23.349 | 4445.32 | 1.4614 | 0.00420 |
| 0.6999 | 1011.61 | -0.629 | 7.479 | 0.378 | 1658.56 | -0.304 | 34.65 | 23.700 | 3578.47 | 1.4649 | 0.00302 |
| 0.8000 | 1008.85 | -0.426 | 7.342 | 0.137 | 1638.97 | -0.175 | 38.32 | 22.062 | 2640.15 | 1.4672 | 0.00163 |
| 0.9000 | 1006.24 | -0.185 | 7.425 | 0.136 | 1622.30 | -0.061 | 39.70 | 15.568 | 1509.07 | 1.4695 | 0.00091 |
| 1.0000 | 1004.58 | 0.000 | 7.359 | 0.000 | 1615.89 | 0.000 | 35.82 | 0.000 | 0.00 | 1.4711 | 0.00000 |
| *313.15* K | | | | | | | | | | | |
| 0.0000 | 992.22 | 0.000 | 3.901 | 0.000 | 1529.42 | 0.000 | 0.61 | 0.000 | 0.00 | 1.3305 | 0.00000 |
| 0.0994 | 1009.57 | -0.375 | 6.607 | 1.314 | 1725.05 | -0.940 | 3.16 | 2.268 | 3853.31 | 1.3900 | 0.00386 |
| 0.1981 | 1016.81 | -0.695 | 7.616 | 1.634 | 1751.15 | -0.998 | 7.44 | 6.138 | 5370.01 | 1.4199 | 0.00620 |
| 0.2979 | 1017.65 | -0.86 | 7.757 | 1.359 | 1733.12 | -0.873 | 12.36 | 10.454 | 5816.30 | 1.4370 | 0.00663 |
| 0.3961 | 1015.87 | -0.900 | 7.688 | 1.016 | 1708.21 | -0.719 | 16.53 | 13.754 | 5611.03 | 1.4475 | 0.00619 |
| 0.4983 | 1013.31 | -0.864 | 7.636 | 0.761 | 1683.20 | -0.564 | 19.63 | 15.526 | 4982.33 | 1.4545 | 0.00510 |
| 0.5987 | 1010.47 | -0.758 | 7.550 | 0.526 | 1661.22 | -0.424 | 23.86 | 17.835 | 4378.55 | 1.4595 | 0.00417 |
| 0.6999 | 1007.82 | -0.616 | 7.520 | 0.381 | 1642.16 | -0.298 | 26.82 | 17.945 | 3518.32 | 1.4629 | 0.00293 |
| 0.8000 | 1005.15 | -0.421 | 7.347 | 0.116 | 1621.35 | -0.166 | 29.92 | 16.906 | 2619.11 | 1.4652 | 0.00157 |
| 0.9000 | 1002.53 | -0.181 | 7.409 | 0.104 | 1605.15 | -0.055 | 31.17 | 12.091 | 1516.01 | 1.4675 | 0.00089 |
| 1.0000 | 1000.89 | 0.000 | 7.368 | 0.000 | 1599.37 | 0.000 | 27.97 | 0.000 | 0.00 | 1.4691 | 0.00000 |

[a] Standard uncertainties u are: $u(T)$ =0.01 K for density, speed of sound and viscosity and $u(T)$ = 0.1 K for refractive index, $u(p)$ = 10 kPa, $u(x_1) = 1\cdot10^{-}$,[4] $u(\rho) = 0.035$ kg·m$^{-3}$, $u(u) = 0.2$ m·s$^{-1}$, $u_r(\eta)$ = 1% , $u(n_D)$ = 0.0002

Table S2. Densities $\rho$, excess molar volumes $V^E$, isobaric thermal expansion coefficients $\alpha_p$ , excess thermal expansion $\Delta\alpha_p$, speeds of sound $u$, excess isentropic compressibilities $k_S^E$, viscosities $\eta$, viscosity deviations $\Delta\eta$, excess Gibbs free energy of activation of viscous flow $\Delta G^{*E}$, refractive indices $n_D$, refractive index deviations $\Delta n_D$ as functions of mole fraction, $x_1$ of DES for TBAC:AP (DES2) + water mixtures at the temperatures (293.15 to 303.15) K and atmospheric pressure. [a]

| $x_1$ | $\rho$ / kg·m$^{-3}$ | $10^6 V_m^E$ / m$^3$·mol$^{-1}$ | $10^4 \alpha_p$ / K$^{-1}$ | $10^4 \Delta\alpha_p$ / K$^{-1}$ | $u$ / m·s$^{-1}$ | $10^{10} k_S^E$ / Pa$^{-1}$ | $\eta$ / mPa·s | $\Delta\eta$ / mPa·s | $\Delta G^{*E}$ / J·mol$^{-1}$ | $n_D$ | $\Delta n_D$ |
|---|---|---|---|---|---|---|---|---|---|---|---|
| 293.15 K | | | | | | | | | | | |
| 0.0000 | 998.20 | 0.000 | 2.134 | 0.000 | 1482.52 | 0.000 | 1.05 | 0.000 | 0.00 | 1.3340 | 0.00000 |
| 0.0982 | 1004.94 | -0.488 | 6.083 | 1.915 | 1788.22 | -1.229 | 6.29 | 4.683 | 3813.83 | 1.3935 | 0.00580 |
| 0.1964 | 1004.60 | -0.843 | 7.265 | 2.062 | 1843.38 | -1.243 | 17.39 | 14.929 | 5494.35 | 1.4225 | 0.00759 |
| 0.2947 | 999.85 | -1.002 | 7.469 | 1.642 | 1831.12 | -1.056 | 30.98 | 27.194 | 5968.10 | 1.4392 | 0.00784 |
| 0.3943 | 994.14 | -1.032 | 7.437 | 1.188 | 1808.34 | -0.856 | 44.48 | 38.604 | 5810.89 | 1.4494 | 0.00690 |
| 0.4899 | 988.81 | -0.974 | 7.407 | 0.866 | 1785.23 | -0.674 | 56.32 | 47.277 | 5324.20 | 1.4563 | 0.00612 |
| 0.5921 | 983.76 | -0.855 | 7.360 | 0.586 | 1763.55 | -0.504 | 65.59 | 51.626 | 4507.89 | 1.4613 | 0.00496 |
| 0.6905 | 979.41 | -0.692 | 7.359 | 0.411 | 1744.52 | -0.356 | 71.07 | 48.982 | 3522.16 | 1.4651 | 0.00416 |
| 0.7946 | 975.37 | -0.483 | 7.351 | 0.255 | 1727.25 | -0.220 | 77.89 | 44.519 | 2461.07 | 1.4679 | 0.00308 |
| 0.8959 | 971.90 | -0.250 | 7.307 | 0.095 | 1713.19 | -0.105 | 85.81 | 33.748 | 1421.25 | 1.4698 | 0.00191 |
| 1.0000 | 968.86 | 0.000 | 7.305 | 0.000 | 1700.32 | 0.000 | 82.77 | 0.000 | 0.00 | 1.4705 | 0.00000 |
| *298.15* K | | | | | | | | | | | |
| 0.0000 | 997.04 | 0.000 | 2.575 | 0.000 | 1496.80 | 0.000 | 0.90 | 0.000 | 0.00 | 1.3331 | 0.00000 |
| 0.0982 | 1001.83 | -0.464 | 6.192 | 1.750 | 1778.69 | -1.161 | 5.06 | 3.705 | 3767.13 | 1.3921 | 0.00550 |
| 0.1964 | 1000.91 | -0.809 | 7.344 | 1.953 | 1827.99 | -1.188 | 13.34 | 11.299 | 5400.17 | 1.4210 | 0.00733 |
| 0.2947 | 996.10 | -0.970 | 7.544 | 1.581 | 1814.97 | -1.019 | 23.10 | 20.014 | 5853.61 | 1.4376 | 0.00757 |
| 0.3943 | 990.43 | -1.005 | 7.508 | 1.158 | 1791.82 | -0.829 | 32.34 | 27.631 | 5672.78 | 1.4478 | 0.00670 |
| 0.4899 | 985.13 | -0.951 | 7.468 | 0.852 | 1768.62 | -0.656 | 40.43 | 33.305 | 5186.02 | 1.4545 | 0.00577 |
| 0.5921 | 980.13 | -0.838 | 7.410 | 0.580 | 1742.83 | -0.476 | 47.04 | 36.223 | 4395.99 | 1.4594 | 0.00455 |
| 0.6905 | 975.80 | -0.678 | 7.396 | 0.406 | 1727.69 | -0.349 | 51.46 | 34.653 | 3457.91 | 1.4631 | 0.00369 |
| 0.7946 | 971.78 | -0.473 | 7.371 | 0.248 | 1710.02 | -0.215 | 56.44 | 31.454 | 2424.21 | 1.4661 | 0.00284 |
| 0.8959 | 968.35 | -0.247 | 7.327 | 0.097 | 1695.62 | -0.102 | 62.41 | 24.102 | 1418.02 | 1.4680 | 0.00169 |
| 1.0000 | 965.32 | 0.000 | 7.315 | 0.000 | 1682.88 | 0.000 | 59.81 | 0.000 | 0.00 | 1.4689 | 0.00000 |
| *303.15* K | | | | | | | | | | | |
| 0.0000 | 995.65 | 0.000 | 3.016 | 0.000 | 1509.53 | 0.000 | 0.79 | 0.000 | 0.00 | 1.3322 | 0.00000 |
| 0.0982 | 998.74 | -0.444 | 6.301 | 1.585 | 1768.06 | -1.095 | 4.20 | 3.029 | 3732.44 | 1.3907 | 0.00528 |
| 0.1964 | 997.19 | -0.777 | 7.422 | 1.844 | 1812.73 | -1.138 | 10.64 | 8.903 | 5333.74 | 1.4194 | 0.00709 |

| | | | | | | | | | | | |
|---|---|---|---|---|---|---|---|---|---|---|---|
| 0.2947 | 992.34 | -0.939 | 7.618 | 1.520 | 1798.92 | -0.984 | 17.89 | 15.305 | 5758.47 | 1.4357 | 0.00715 |
| 0.3943 | 986.70 | -0.978 | 7.578 | 1.129 | 1775.40 | -0.805 | 24.43 | 20.548 | 5550.79 | 1.4460 | 0.00647 |
| 0.4899 | 981.45 | -0.928 | 7.529 | 0.838 | 1751.99 | -0.638 | 30.08 | 24.297 | 5055.75 | 1.4527 | 0.00560 |
| 0.5921 | 976.49 | -0.820 | 7.460 | 0.575 | 1726.03 | -0.463 | 35.17 | 26.529 | 4305.18 | 1.4576 | 0.00443 |
| 0.6905 | 972.20 | -0.664 | 7.432 | 0.402 | 1710.85 | -0.341 | 38.66 | 25.456 | 3402.54 | 1.4612 | 0.00351 |
| 0.7946 | 968.20 | -0.463 | 7.392 | 0.241 | 1693.30 | -0.211 | 42.88 | 23.544 | 2420.68 | 1.4640 | 0.00249 |
| 0.8959 | 964.81 | -0.242 | 7.348 | 0.100 | 1678.51 | -0.099 | 47.33 | 18.160 | 1433.04 | 1.4658 | 0.00127 |
| 1.0000 | 961.80 | 0.000 | 7.325 | 0.000 | 1665.99 | 0.000 | 44.78 | 0.000 | 0.00 | 1.4671 | 0.00000 |
| *308.15* K | | | | | | | | | | | |
| 0.0000 | 994.03 | 0.000 | 3.457 | 0.000 | 1520.29 | 0.000 | 0.69 | 0.000 | 0.00 | 1.3314 | 0.00000 |
| 0.0982 | 995.58 | -0.425 | 6.410 | 1.421 | 1757.38 | -1.039 | 3.67 | 2.661 | 3828.70 | 1.3897 | 0.00505 |
| 0.1964 | 993.45 | -0.747 | 7.500 | 1.735 | 1797.30 | -1.094 | 8.70 | 7.222 | 5322.88 | 1.4184 | 0.00688 |
| 0.2947 | 988.55 | -0.909 | 7.693 | 1.460 | 1782.62 | -0.953 | 14.2 | 12.029 | 5713.07 | 1.4347 | 0.00696 |
| 0.3943 | 982.95 | -0.951 | 7.648 | 1.100 | 1758.52 | -0.782 | 18.92 | 15.703 | 5472.45 | 1.4448 | 0.00610 |
| 0.4899 | 977.75 | -0.906 | 7.590 | 0.824 | 1735.23 | -0.623 | 23.22 | 18.491 | 4993.20 | 1.4515 | 0.00525 |
| 0.5921 | 972.85 | -0.802 | 7.510 | 0.570 | 1708.88 | -0.451 | 27.45 | 20.476 | 4292.79 | 1.4563 | 0.00399 |
| 0.6905 | 968.58 | -0.650 | 7.468 | 0.398 | 1693.99 | -0.334 | 30.54 | 20.032 | 3439.23 | 1.4599 | 0.00308 |
| 0.7946 | 964.63 | -0.454 | 7.413 | 0.234 | 1676.43 | -0.207 | 33.23 | 18.034 | 2427.03 | 1.4627 | 0.00207 |
| 0.8959 | 961.26 | -0.238 | 7.368 | 0.102 | 1661.65 | -0.098 | 35.89 | 13.275 | 1401.48 | 1.4646 | 0.00096 |
| 1.0000 | 958.28 | 0.000 | 7.336 | 0.000 | 1648.89 | 0.000 | 34.23 | 0.000 | 0.00 | 1.4662 | 0.00000 |
| *313.15* K | | | | | | | | | | | |
| 0.0000 | 992.21 | 0.000 | 3.898 | 0.000 | 1529.42 | 0.000 | 0.61 | 0.000 | 0.00 | 1.3305 | 0.00000 |
| 0.0982 | 992.34 | -0.408 | 6.519 | 1.258 | 1746.33 | -0.987 | 3.14 | 2.259 | 3841.10 | 1.3882 | 0.00483 |
| 0.1964 | 989.68 | -0.718 | 7.578 | 1.627 | 1781.41 | -1.052 | 7.43 | 6.156 | 5388.88 | 1.4167 | 0.00667 |
| 0.2947 | 984.73 | -0.880 | 7.767 | 1.401 | 1766.25 | -0.925 | 11.33 | 9.482 | 5642.12 | 1.4328 | 0.00670 |
| 0.3943 | 979.18 | -0.926 | 7.718 | 1.071 | 1741.86 | -0.762 | 15.00 | 12.297 | 5412.64 | 1.4428 | 0.00584 |
| 0.4899 | 974.03 | -0.883 | 7.651 | 0.811 | 1718.48 | -0.609 | 17.74 | 13.816 | 4860.83 | 1.4495 | 0.00506 |
| 0.5921 | 969.19 | -0.785 | 7.560 | 0.566 | 1692.08 | -0.441 | 21.26 | 15.547 | 4218.20 | 1.4543 | 0.00386 |
| 0.6905 | 964.96 | -0.637 | 7.504 | 0.394 | 1676.87 | -0.327 | 24.19 | 15.697 | 3441.56 | 1.4577 | 0.00279 |
| 0.7946 | 961.05 | -0.445 | 7.434 | 0.227 | 1659.64 | -0.204 | 26.42 | 14.286 | 2456.99 | 1.4604 | 0.00172 |
| 0.8959 | 957.72 | -0.233 | 7.389 | 0.104 | 1644.80 | -0.096 | 28.01 | 10.187 | 1399.79 | 1.4623 | 0.00064 |
| 1.0000 | 954.77 | 0.000 | 7.346 | 0.000 | 1631.99 | 0.000 | 26.61 | 0.000 | 0.00 | 1.4642 | 0.00000 |

[a] Standard uncertainties u are: $u(T)$ =0.01 K for density, speed of sound and viscosity and $u(T)$ = 0.1 K for refractive index, $u(p)$ = 10 kPa, $u(x_1) = 1\cdot10^{-}$,[4] $u(\rho) = 0.035$ kg·m$^{-3}$, $u(u) = 0.2$ m·s$^{-1}$, $u_r(\eta)$ = 1% , $u(n_D)$ = 0.0002

Table S3. Densities $\rho$, excess molar volumes $V^E$, isobaric thermal expansion coefficients $\alpha_p$ , excess thermal expansion $\Delta\alpha_p$, speed of sounds $u$, excess isentropic compressibilities $k_S^E$, viscosities $\eta$, viscosity deviations $\Delta\eta$, excess Gibbs free energy of activation of viscous flow $\Delta G^{*E}$, refractive indices $n_D$, refractive index deviations $\Delta n_D$ as functions of mole fraction, $x_1$ of DES for TBAB:MAE (DES3) + water mixtures at the temperatures (293.15 to 303.15) K and atmospheric pressure. [a]

| $x_1$ | $\rho$ / kg·m$^{-3}$ | $10^6 V_m^E$ / m$^3$·mol$^{-1}$ | $10^4\ \alpha_p$ / K$^{-1}$ | $10^4\Delta\alpha_p$ / K$^{-1}$ | $u$ / m·s$^{-1}$ | $10^{10}\ k_S^E$ / Pa$^{-1}$ | $\eta$ / mPa·s | $\Delta\eta$ / mPa·s | $\Delta G^{*E}$ / J·mol$^{-1}$ | $n_D$ | $\Delta n_D$ |
|---|---|---|---|---|---|---|---|---|---|---|---|
| 293.15 K | | | | | | | | | | | |
| 0.0000 | 998.20 | 0.000 | 2.119 | 0.000 | 1482.52 | 0.000 | 1.05 | 0.000 | 0.00 | 1.3340 | 0.00000 |
| 0.0978 | 1016.19 | -0.617 | 6.186 | 1.965 | 1738.92 | -1.463 | 6.76 | 5.235 | 4147.73 | 1.3921 | 0.00631 |
| 0.1982 | 1018.89 | -1.017 | 7.364 | 2.083 | 1731.41 | -1.462 | 18.26 | 16.039 | 5912.10 | 1.4208 | 0.00888 |
| 0.2957 | 1014.71 | -1.155 | 7.383 | 1.489 | 1686.27 | -1.251 | 28.68 | 25.463 | 6229.61 | 1.4360 | 0.00899 |
| 0.3970 | 1009.27 | -1.159 | 7.375 | 1.060 | 1641.46 | -1.009 | 39.11 | 34.394 | 6071.66 | 1.4452 | 0.00780 |
| 0.4958 | 1004.09 | -1.072 | 7.323 | 0.716 | 1604.42 | -0.786 | 45.73 | 38.886 | 5492.02 | 1.4506 | 0.00602 |
| 0.5935 | 999.33 | -0.917 | 7.280 | 0.457 | 1574.10 | -0.587 | 50.05 | 40.149 | 4711.38 | 1.4543 | 0.00439 |
| 0.6956 | 995.01 | -0.713 | 7.264 | 0.266 | 1548.23 | -0.404 | 51.50 | 36.934 | 3692.97 | 1.4572 | 0.00298 |
| 0.7979 | 991.43 | -0.494 | 7.279 | 0.141 | 1527.38 | -0.248 | 51.65 | 30.199 | 2575.62 | 1.4594 | 0.00175 |
| 0.8940 | 988.31 | -0.248 | 7.301 | 0.057 | 1510.73 | -0.117 | 51.65 | 20.805 | 1495.23 | 1.4613 | 0.00101 |
| 1.0000 | 985.67 | 0.000 | 7.342 | 0.000 | 1496.77 | 0.000 | 46.05 | 0.000 | 0.00 | 1.4627 | 0.00000 |
| *298.15* K | | | | | | | | | | | |
| 0.0000 | 997.05 | 0.000 | 2.565 | 0.000 | 1496.80 | 0.000 | 0.90 | 0.000 | 0.00 | 1.3331 | 0.00000 |
| 0.0978 | 1013.00 | -0.593 | 6.341 | 1.839 | 1727.94 | -1.399 | 5.35 | 4.064 | 4059.50 | 1.3905 | 0.00598 |
| 0.1982 | 1015.12 | -0.984 | 7.459 | 1.981 | 1717.11 | -1.420 | 13.77 | 11.911 | 5765.79 | 1.4190 | 0.00853 |
| 0.2957 | 1010.94 | -1.125 | 7.483 | 1.441 | 1671.56 | -1.224 | 21.14 | 18.495 | 6065.45 | 1.4342 | 0.00874 |
| 0.3970 | 1005.53 | -1.135 | 7.465 | 1.036 | 1626.62 | -0.992 | 28.75 | 24.922 | 5931.15 | 1.4433 | 0.00752 |
| 0.4958 | 1000.40 | -1.053 | 7.403 | 0.706 | 1589.56 | -0.776 | 33.30 | 27.813 | 5350.14 | 1.4487 | 0.00579 |
| 0.5935 | 995.67 | -0.903 | 7.355 | 0.459 | 1559.06 | -0.580 | 36.52 | 28.685 | 4593.14 | 1.4524 | 0.00420 |
| 0.6956 | 991.38 | -0.705 | 7.327 | 0.271 | 1533.11 | -0.401 | 38.12 | 26.751 | 3626.27 | 1.4553 | 0.00282 |
| 0.7979 | 987.82 | -0.490 | 7.331 | 0.146 | 1512.16 | -0.246 | 38.33 | 21.820 | 2530.01 | 1.4575 | 0.00161 |
| 0.8940 | 984.70 | -0.247 | 7.344 | 0.062 | 1495.39 | -0.116 | 38.31 | 14.869 | 1461.16 | 1.4594 | 0.00089 |
| 1.0000 | 982.05 | 0.000 | 7.372 | 0.000 | 1481.33 | 0.000 | 34.50 | 0.000 | 0.00 | 1.4609 | 0.00000 |
| *303.15* K | | | | | | | | | | | |
| 0.0000 | 994.03 | 0.000 | 3.010 | 0.000 | 1509.53 | 0.000 | 0.79 | 0.000 | 0.00 | 1.3322 | 0.00000 |
| 0.0978 | 1006.45 | -0.571 | 6.496 | 1.713 | 1716.80 | -1.341 | 4.42 | 3.300 | 4006.30 | 1.3889 | 0.00568 |
| 0.1982 | 1007.48 | -0.952 | 7.555 | 1.880 | 1702.63 | -1.382 | 10.86 | 9.267 | 5658.63 | 1.4172 | 0.00823 |

| | | | | | | | | | | | |
|---|---|---|---|---|---|---|---|---|---|---|---|
| 0.2957 | 1003.31 | -1.097 | 7.582 | 1.394 | 1656.36 | -1.200 | 16.31 | 14.066 | 5937.07 | 1.4323 | 0.00846 |
| 0.3970 | 997.97 | -1.110 | 7.556 | 1.013 | 1611.59 | -0.978 | 21.90 | 18.700 | 5799.91 | 1.4414 | 0.00732 |
| 0.4958 | 992.94 | -1.034 | 7.484 | 0.697 | 1574.83 | -0.769 | 25.07 | 20.533 | 5209.02 | 1.4467 | 0.00555 |
| 0.5935 | 988.31 | -0.889 | 7.430 | 0.462 | 1543.96 | -0.575 | 27.41 | 21.017 | 4461.74 | 1.4504 | 0.00400 |
| 0.6956 | 984.08 | -0.696 | 7.391 | 0.276 | 1517.78 | -0.397 | 28.15 | 18.988 | 3468.73 | 1.4533 | 0.00265 |
| 0.7979 | 980.55 | -0.484 | 7.383 | 0.152 | 1496.98 | -0.245 | 28.83 | 15.692 | 2431.52 | 1.4555 | 0.00147 |
| 0.8940 | 977.46 | -0.244 | 7.387 | 0.066 | 1480.08 | -0.115 | 29.47 | 11.036 | 1430.64 | 1.4574 | 0.00078 |
| 1.0000 | 974.81 | 0.000 | 7.403 | 0.000 | 1465.91 | 0.000 | 26.78 | 0.000 | 0.00 | 1.4590 | 0.00000 |
| *308.15* K | | | | | | | | | | | |
| 0.0000 | 994.03 | 0.000 | 3.455 | 0.000 | 1520.29 | 0.000 | 0.69 | 0.000 | 0.00 | 1.3314 | 0.00000 |
| 0.0978 | 1006.45 | -0.550 | 6.651 | 1.588 | 1705.28 | -1.291 | 3.73 | 2.761 | 4013.42 | 1.3876 | 0.00542 |
| 0.1982 | 1007.48 | -0.922 | 7.650 | 1.780 | 1688.00 | -1.349 | 8.78 | 7.421 | 5612.26 | 1.4157 | 0.00790 |
| 0.2957 | 1003.31 | -1.069 | 7.682 | 1.347 | 1641.36 | -1.180 | 12.76 | 10.858 | 5835.10 | 1.4308 | 0.00819 |
| 0.3970 | 997.97 | -1.087 | 7.646 | 0.990 | 1596.47 | -0.967 | 17.17 | 14.483 | 5726.92 | 1.4399 | 0.00711 |
| 0.4958 | 992.94 | -1.016 | 7.564 | 0.688 | 1559.75 | -0.763 | 20.22 | 16.460 | 5225.68 | 1.4452 | 0.00536 |
| 0.5935 | 988.31 | -0.875 | 7.505 | 0.465 | 1528.83 | -0.571 | 21.73 | 16.479 | 4446.77 | 1.4489 | 0.00384 |
| 0.6956 | 984.08 | -0.686 | 7.454 | 0.281 | 1502.71 | -0.395 | 22.37 | 14.924 | 3469.99 | 1.4518 | 0.00251 |
| 0.7979 | 980.55 | -0.478 | 7.435 | 0.157 | 1481.63 | -0.243 | 22.88 | 12.315 | 2438.83 | 1.4540 | 0.00135 |
| 0.8940 | 977.46 | -0.241 | 7.430 | 0.071 | 1464.88 | -0.115 | 23.35 | 8.673 | 1442.26 | 1.4559 | 0.00066 |
| 1.0000 | 974.81 | 0.000 | 7.433 | 0.000 | 1450.59 | 0.000 | 21.09 | 0.000 | 0.00 | 1.4576 | 0.00000 |
| *313.15* K | | | | | | | | | | | |
| 0.0000 | 992.22 | 0.000 | 3.901 | 0.000 | 1529.42 | 0.000 | 0.61 | 0.000 | 0.00 | 1.3305 | 0.00000 |
| 0.0978 | 1003.07 | -0.531 | 6.806 | 1.464 | 1693.90 | -1.247 | 3.18 | 2.331 | 4011.77 | 1.3861 | 0.00518 |
| 0.1982 | 1003.61 | -0.893 | 7.746 | 1.681 | 1672.92 | -1.319 | 7.25 | 6.072 | 5580.17 | 1.4140 | 0.00764 |
| 0.2957 | 999.43 | -1.043 | 7.782 | 1.300 | 1626.16 | -1.163 | 10.25 | 8.616 | 5758.91 | 1.4289 | 0.00784 |
| 0.3970 | 994.13 | -1.063 | 7.736 | 0.968 | 1581.28 | -0.957 | 13.63 | 11.349 | 5644.25 | 1.4381 | 0.00693 |
| 0.4958 | 989.17 | -0.997 | 7.645 | 0.679 | 1544.61 | -0.757 | 15.94 | 12.772 | 5140.47 | 1.4433 | 0.00514 |
| 0.5935 | 984.58 | -0.861 | 7.580 | 0.468 | 1514.04 | -0.570 | 17.07 | 12.688 | 4363.91 | 1.4470 | 0.00365 |
| 0.6956 | 980.41 | -0.677 | 7.517 | 0.286 | 1487.62 | -0.394 | 17.33 | 11.178 | 3360.36 | 1.4499 | 0.00235 |
| 0.7979 | 976.90 | -0.473 | 7.487 | 0.163 | 1466.54 | -0.243 | 17.86 | 9.218 | 2357.62 | 1.4521 | 0.00121 |
| 0.8940 | 973.82 | -0.238 | 7.473 | 0.076 | 1449.56 | -0.113 | 18.17 | 6.276 | 1360.62 | 1.4540 | 0.00055 |
| 1.0000 | 971.19 | 0.000 | 7.463 | 0.000 | 1435.41 | 0.000 | 16.92 | 0.000 | 0.00 | 1.4558 | 0.00000 |

[a] Standard uncertainties u are: $u(T)$ =0.01 K for density, speed of sound and viscosity and $u(T)$ = 0.1 K for refractive index, $u(p)$ = 10 kPa, $u(x_1) = 1\cdot 10^{-}$,$^{4}$ $u(\rho) = 0.035$ kg·m$^{-3}$, $u(u) = 0.2$ m·s$^{-1}$, $u_r(\eta)$ = 1% , $u(n_D)$ = 0.0002

Table S4. Densities $\rho$, excess molar volumes $V^E$, isobaric thermal expansion coefficients $\alpha_p$ , excess thermal expansion $\Delta\alpha_p$, speed of sound $u$, excess isentropic compressibilities $k_S^E$, viscosities $\eta$, viscosity deviations $\Delta\eta$, excess Gibbs free energy of activation of viscous flow $\Delta G^{*E}$, refractive indices $n_D$, refractive index deviations $\Delta n_D$ as functions of mole fraction, $x_1$ of DES for TBAB:BAE (DES4) + water mixtures at the temperatures (293.15 to 303.15) K and atmospheric pressure. [a]

| $x_1$ | $\rho$ / kg·m$^{-3}$ | $10^6 V_m^E$ / m$^3$·mol$^{-1}$ | $10^4\ \alpha_p$ / K$^{-1}$ | $10^4 \Delta\alpha_p$ / K$^{-1}$ | $u$ / m·s$^{-1}$ | $10^{10}\ k_S^E$ / Pa$^{-1}$ | $\eta$ / mPa·s | $\Delta\eta$ / mPa·s | $\Delta G^{*E}$ / J·mol$^{-1}$ | $n_D$ | $\Delta n_D$ |
|---|---|---|---|---|---|---|---|---|---|---|---|
| 293.15 K | | | | | | | | | | | |
| 0.0000 | 998.20 | 0.000 | 2.119 | 0.000 | 1482.52 | 0.000 | 1.05 | 0.000 | 0.00 | 1.334 | 0.00000 |
| 0.0975 | 987.04 | -0.583 | 6.633 | 1.805 | 1605.54 | -1.196 | 8.64 | 7.086 | 4982.17 | 1.3999 | 0.00554 |
| 0.1951 | 976.01 | -0.879 | 7.459 | 1.543 | 1580.95 | -1.160 | 20.06 | 17.758 | 6430.49 | 1.4261 | 0.00750 |
| 0.2936 | 967.54 | -1.030 | 7.684 | 1.177 | 1548.49 | -0.980 | 33.26 | 29.838 | 6827.16 | 1.4391 | 0.00735 |
| 0.3943 | 960.63 | -1.069 | 7.754 | 0.874 | 1521.14 | -0.791 | 46.71 | 41.580 | 6664.02 | 1.4464 | 0.00631 |
| 0.4930 | 955.07 | -1.019 | 7.753 | 0.624 | 1499.17 | -0.620 | 54.37 | 46.739 | 5974.18 | 1.4509 | 0.00525 |
| 0.5925 | 950.31 | -0.896 | 7.738 | 0.426 | 1480.24 | -0.461 | 60.92 | 49.533 | 5120.29 | 1.4537 | 0.00399 |
| 0.6975 | 946.07 | -0.708 | 7.726 | 0.269 | 1463.88 | -0.314 | 62.40 | 45.023 | 3933.83 | 1.4558 | 0.00286 |
| 0.7920 | 942.81 | -0.502 | 7.731 | 0.171 | 1451.18 | -0.196 | 62.48 | 37.068 | 2782.20 | 1.4573 | 0.00208 |
| 0.8938 | 939.77 | -0.247 | 7.718 | 0.069 | 1440.66 | -0.091 | 60.29 | 22.015 | 1421.51 | 1.4585 | 0.00129 |
| 1.0000 | 937.31 | 0.000 | 7.725 | 0.000 | 1431.73 | 0.000 | 58.66 | 0.000 | 0.00 | 1.4589 | 0.00000 |
| *298.15* K | | | | | | | | | | | |
| 0.0000 | 997.05 | 0.000 | 2.565 | 0.000 | 1496.80 | 0.000 | 0.90 | 0.000 | 0.00 | 1.3331 | 0.00000 |
| 0.0975 | 983.73 | -0.557 | 6.769 | 1.693 | 1596.27 | -1.152 | 6.85 | 5.536 | 4912.72 | 1.3984 | 0.00537 |
| 0.1951 | 972.34 | -0.847 | 7.570 | 1.488 | 1567.74 | -1.132 | 15.28 | 13.361 | 6321.05 | 1.4244 | 0.00731 |
| 0.2936 | 963.80 | -0.999 | 7.793 | 1.165 | 1533.63 | -0.961 | 25.00 | 22.181 | 6725.16 | 1.4372 | 0.00705 |
| 0.3943 | 956.88 | -1.041 | 7.871 | 0.896 | 1505.66 | -0.778 | 34.26 | 30.101 | 6533.68 | 1.4445 | 0.00608 |
| 0.4930 | 951.35 | -0.996 | 7.837 | 0.633 | 1483.43 | -0.611 | 39.21 | 33.109 | 5824.06 | 1.4490 | 0.00506 |
| 0.5925 | 946.62 | -0.878 | 7.811 | 0.438 | 1464.64 | -0.457 | 43.81 | 34.829 | 4982.49 | 1.4518 | 0.00382 |
| 0.6975 | 942.40 | -0.695 | 7.787 | 0.279 | 1448.91 | -0.318 | 45.11 | 31.617 | 3825.30 | 1.4539 | 0.00271 |
| 0.7920 | 939.16 | -0.493 | 7.776 | 0.174 | 1435.02 | -0.192 | 45.50 | 26.028 | 2704.65 | 1.4554 | 0.00195 |
| 0.8938 | 936.14 | -0.243 | 7.757 | 0.073 | 1424.82 | -0.092 | 44.27 | 15.354 | 1375.95 | 1.4565 | 0.00108 |
| 1.0000 | 933.69 | 0.000 | 7.754 | 0.000 | 1415.72 | 0.000 | 43.66 | 0.000 | 0.00 | 1.4571 | 0.00000 |
| *303.15* K | | | | | | | | | | | |
| 0.0000 | 995.65 | 0.000 | 3.010 | 0.000 | 1509.53 | 0.000 | 0.79 | 0.000 | 0.00 | 1.3322 | 0.00000 |
| 0.0975 | 980.38 | -0.534 | 6.904 | 1.581 | 1587.12 | -1.117 | 5.65 | 4.507 | 4874.04 | 1.3969 | 0.00526 |
| 0.1951 | 968.65 | -0.818 | 7.681 | 1.433 | 1553.86 | -1.106 | 12.20 | 10.555 | 6259.26 | 1.4226 | 0.00709 |

| | | | | | | | | | | | |
|---|---|---|---|---|---|---|---|---|---|---|---|
| 0.2936 | 960.03 | -0.969 | 7.903 | 1.153 | 1518.73 | -0.945 | 19.53 | 17.157 | 6650.31 | 1.4353 | 0.00684 |
| 0.3943 | 953.10 | -1.012 | 7.987 | 0.920 | 1490.41 | -0.768 | 25.84 | 22.381 | 6400.73 | 1.4425 | 0.00583 |
| 0.4930 | 947.61 | -0.973 | 7.921 | 0.642 | 1467.70 | -0.604 | 29.54 | 24.534 | 5709.66 | 1.4470 | 0.00485 |
| 0.5925 | 942.91 | -0.859 | 7.885 | 0.451 | 1449.33 | -0.456 | 32.86 | 25.589 | 4876.35 | 1.4498 | 0.00365 |
| 0.6975 | 938.73 | -0.682 | 7.847 | 0.290 | 1432.25 | -0.309 | 33.81 | 23.043 | 3733.45 | 1.4519 | 0.00256 |
| 0.7920 | 935.51 | -0.483 | 7.822 | 0.178 | 1418.25 | -0.184 | 34.21 | 18.867 | 2633.52 | 1.4534 | 0.00182 |
| 0.8938 | 932.51 | -0.238 | 7.796 | 0.077 | 1408.61 | -0.088 | 33.47 | 11.005 | 1331.17 | 1.4545 | 0.00096 |
| 1.0000 | 930.07 | 0.000 | 7.783 | 0.000 | 1399.85 | 0.000 | 33.43 | 0.000 | 0.00 | 1.4552 | 0.00000 |
| *308.15* K | | | | | | | | | | | |
| 0.0000 | 994.03 | 0.000 | 3.455 | 0.000 | 1520.29 | 0.000 | 0.69 | 0.000 | 0.00 | 1.3314 | 0.00000 |
| 0.0975 | 976.97 | -0.511 | 7.040 | 1.471 | 1577.06 | -1.084 | 4.69 | 3.708 | 4861.36 | 1.3954 | 0.00514 |
| 0.1951 | 964.91 | -0.789 | 7.793 | 1.379 | 1539.85 | -1.082 | 9.91 | 8.511 | 6243.95 | 1.4208 | 0.00690 |
| 0.2936 | 956.21 | -0.939 | 8.012 | 1.141 | 1503.75 | -0.930 | 15.55 | 13.552 | 6620.04 | 1.4333 | 0.00657 |
| 0.3943 | 949.28 | -0.982 | 8.104 | 0.943 | 1474.09 | -0.753 | 20.44 | 17.562 | 6380.94 | 1.4405 | 0.00565 |
| 0.4930 | 943.84 | -0.948 | 8.005 | 0.652 | 1451.90 | -0.596 | 22.94 | 18.826 | 5661.78 | 1.4449 | 0.00463 |
| 0.5925 | 939.19 | -0.839 | 7.958 | 0.464 | 1433.59 | -0.451 | 25.10 | 19.196 | 4803.08 | 1.4477 | 0.00346 |
| 0.6975 | 935.04 | -0.667 | 7.908 | 0.301 | 1416.38 | -0.305 | 25.76 | 17.131 | 3667.46 | 1.4498 | 0.00241 |
| 0.7920 | 931.85 | -0.474 | 7.867 | 0.182 | 1402.68 | -0.183 | 26.39 | 14.240 | 2610.80 | 1.4513 | 0.00168 |
| 0.8938 | 928.87 | -0.234 | 7.835 | 0.081 | 1392.95 | -0.087 | 25.82 | 8.252 | 1318.63 | 1.4524 | 0.00085 |
| 1.0000 | 926.45 | 0.000 | 7.813 | 0.000 | 1384.17 | 0.000 | 25.81 | 0.000 | 0.00 | 1.4532 | 0.00000 |
| *313.15* K | | | | | | | | | | | |
| 0.0000 | 992.22 | 0.000 | 3.901 | 0.000 | 1529.42 | 0.000 | 0.61 | 0.000 | 0.00 | 1.3305 | 0.00000 |
| 0.0975 | 973.50 | -0.490 | 7.176 | 1.681 | 1566.84 | -1.057 | 3.92 | 3.062 | 4827.81 | 1.3941 | 0.00493 |
| 0.1951 | 961.13 | -0.761 | 7.904 | 1.609 | 1526.93 | -1.069 | 8.14 | 6.932 | 6217.66 | 1.4194 | 0.00667 |
| 0.2936 | 952.37 | -0.909 | 8.122 | 1.366 | 1488.46 | -0.915 | 12.92 | 11.208 | 6658.46 | 1.4319 | 0.00639 |
| 0.3943 | 945.41 | -0.950 | 8.221 | 1.148 | 1459.07 | -0.747 | 16.24 | 13.811 | 6330.27 | 1.4391 | 0.00549 |
| 0.4930 | 940.05 | -0.923 | 8.089 | 0.797 | 1436.26 | -0.591 | 17.98 | 14.546 | 5593.38 | 1.4434 | 0.00438 |
| 0.5925 | 935.44 | -0.819 | 8.032 | 0.578 | 1417.33 | -0.444 | 19.50 | 14.634 | 4727.97 | 1.4462 | 0.00323 |
| 0.6975 | 931.33 | -0.652 | 7.968 | 0.384 | 1400.79 | -0.304 | 20.08 | 13.044 | 3612.81 | 1.4483 | 0.00219 |
| 0.7920 | 928.18 | -0.464 | 7.913 | 0.224 | 1388.11 | -0.190 | 20.42 | 10.629 | 2548.67 | 1.4499 | 0.00157 |
| 0.8938 | 925.23 | -0.229 | 7.874 | 0.105 | 1377.38 | -0.087 | 20.29 | 6.295 | 1305.52 | 1.4510 | 0.00074 |
| 1.0000 | 922.83 | 0.000 | 7.842 | 0.000 | 1368.57 | 0.000 | 20.30 | 0.000 | 0.00 | 1.4519 | 0.00000 |

[a] Standard uncertainties u are: $u(T)$ =0.01 K for density, speed of sound and viscosity and $u(T)$ = 0.1 K for refractive index, $u(p)$ = 10 kPa, $u(x_1) = 1\cdot10^{-}$,$^{4}$ $u(\rho)$ = 0.035 $kg\cdot m^{-3}$, $u(u)$ = 0.2 $m\cdot s^{-1}$, $u_r(\eta)$ = 1% , $u(n_D)$ = 0.0002

Table S5 Parameters of the JAM equation, together with *RMSD* and *ARD%* for density, speed of sound, viscosity and refractive index of DES (1) + water (2) systems at different temperatures.

| $T$ / K | 293.15 | 298.15 | 303.15 | 308.15 | 313.15 | 293.15 | 298.15 | 303.15 | 308.15 | 313.15 |
|---|---|---|---|---|---|---|---|---|---|---|
| | | | | | $\rho$ / kg·m$^{-3}$ | | | | | |
| | | | DES1 (1) + water (2) | | | | | DES2 (1) + water (2) | | |
| $A_0$ | 24.8275 | 23.7395 | 22.6995 | 21.7383 | 20.8103 | 5.7143 | 4.2731 | 2.8780 | 1.5468 | 0.2672 |
| $A_1$ | -27.0200 | -25.8625 | -24.7000 | -23.6118 | -22.6113 | -13.9849 | -12.6833 | -11.3833 | -10.1036 | -8.8636 |
| $A_2$ | 26.7075 | 24.7500 | 23.0350 | 21.3838 | 19.9113 | 16.3915 | 14.9069 | 13.3060 | 11.7903 | 10.3483 |
| $A_3$ | -17.9650 | -16.4675 | -15.1600 | -13.9238 | -12.6038 | -9.7227 | -7.6083 | -5.9668 | -4.4809 | -3.0999 |
| $A_4$ | - | - | - | - | - | -0.5512 | -1.9659 | -2.6380 | -3.2256 | -3.7564 |
| $R^2$ | 0.9999 | 0.9999 | 0.9999 | 0.9999 | 0.9999 | 0.9996 | 0.9994 | 0.9993 | 0.9992 | 0.9990 |
| *RMSD* | 0.00017 | 0.00016 | 0.00013 | 0.00013 | 0.00013 | 0.00014 | 0.00014 | 0.00013 | 0.00011 | 0.00010 |
| *ARD%* | 0.0094 | 0.0086 | 0.0068 | 0.0063 | 0.0064 | 0.0073 | 0.0074 | 0.0067 | 0.0060 | 0.0054 |
| | | | DES3 (1) + water (2) | | | | | DES4 (1) + water (2) | | |
| $A_0$ | 13.5545 | 12.3320 | 11.1835 | 10.0645 | 8.9810 | -15.2749 | -17.2111 | -19.0931 | -20.9427 | -22.7449 |
| $A_1$ | -21.6575 | -20.4875 | -19.3300 | -18.2375 | -17.1650 | 6.3675 | 7.8770 | 9.3910 | 10.8915 | 12.4003 |
| $A_2$ | 29.3125 | 27.2200 | 25.2725 | 23.4350 | 21.7125 | -4.3187 | -5.4068 | -6.4975 | -7.5559 | -8.5685 |
| $A_3$ | -23.8975 | -21.7775 | -19.915 | -18.1625 | -16.5600 | -4.6725 | -1.7230 | 0.9155 | 3.3840 | 5.6678 |
| $A_4$ | - | - | - | - | - | 7.7956 | 4.6164 | 1.7841 | -0.8624 | -3.3666 |
| $R^2$ | 0.9991 | 0.9992 | 0.9993 | 0.9993 | 0.9994 | 0.9995 | 0.9998 | 0.9995 | 0.9999 | 0.9999 |
| *RMSD* | 0.00037 | 0.00032 | 0.00027 | 0.00024 | 0.00020 | 0.00016 | 0.00011 | 0.00008 | 0.00005 | 0.00004 |
| *ARD%* | 0.020 | 0.017 | 0.015 | 0.012 | 0.010 | 0.0087 | 0.0062 | 0.0039 | 0.0026 | 0.0018 |
| | | | | | $u$ / m·s$^{-1}$ | | | | | |
| | | | DES1 (1) + water (2) | | | | | DES2 (1) + water (2) | | |
| $A_0$ | 126.286 | 116.013 | 108.149 | 100.740 | 92.017 | 136.992 | 128.015 | 120.735 | 111.169 | 103.579 |
| $A_1$ | -127.313 | -121.125 | -113.488 | -106.725 | -101.388 | -138.025 | -133.000 | -126.000 | -121.438 | -115.475 |
| $A_2$ | 114.750 | 112.000 | 112.450 | 106.125 | 99.588 | 126.350 | 125.250 | 122.000 | 117.500 | 112.213 |
| $A_3$ | -258.188 | -243.875 | -228.613 | -214.475 | -201.663 | -263.275 | -242.500 | -225.000 | -209.063 | -193.475 |

| | | | | | | | | | | |
|---|---|---|---|---|---|---|---|---|---|---|
| $A_4$ | 234.594 | 206.188 | 177.731 | 161.500 | 145.525 | 248.038 | 225.250 | 203.875 | 186.656 | 172.494 |
| $R^2$ | 0.9994 | 0.9994 | 0.9994 | 0.9994 | 0.9995 | 0.9995 | 0.9992 | 0.9993 | 0.9992 | 0.9992 |
| *RMSD* | 0.0025 | 0.0024 | 0.0020 | 0.0022 | 0.0016 | 0.0023 | 0.0026 | 0.0034 | 0.0023 | 0.0021 |
| *%ARD* | 0.14 | 0.13 | 0.11 | 0.12 | 0.09 | 0.12 | 0.15 | 0.19 | 0.13 | 0.12 |
| | | DES3 (1) + water (2) | | | | | DES4 (1) + water (2) | | | |
| $A_0$ | 86.371 | 79.321 | 67.809 | 59.512 | 51.428 | 34.050 | 22.706 | 11.316 | 0.518 | -9.699 |
| $A_1$ | -106.713 | -98.563 | -93.988 | -87.900 | -81.000 | -41.275 | -32.387 | -25.325 | -16.862 | -9.175 |
| $A_2$ | 96.600 | 93.250 | 87.200 | 82.975 | 77.875 | 28.975 | 25.025 | 14.037 | 9.713 | 9.487 |
| $A_3$ | -293.088 | -272.938 | -254.113 | -236.400 | -221.500 | -179.525 | -162.913 | -146.125 | -130.338 | -113.775 |
| $A_4$ | 307.944 | 284.219 | 264.731 | 244.288 | 227.063 | 213.100 | 189.619 | 177.631 | 158.275 | 135.331 |
| $R^2$ | 0.9981 | 0.9982 | 0.9983 | 0.9983 | 0.9983 | 0.9957 | 0.9955 | 0.9951 | 0.9948 | 0.9946 |
| *RMSD* | 0.0044 | 0.0044 | 0.0039 | 0.003 | 0.0027 | 0.0032 | 0.0027 | 0.0024 | 0.0020 | 0.0017 |
| *%ARD* | 0.22 | 0.22 | 0.20 | 0.15 | 0.13 | 0.16 | 0.14 | 0.12 | 0.10 | 0.08 |
| | | | | | $\eta$ / mPa·s | | | | | |
| | | DES1 (1) + water (2) | | | | | DES2 (1) + water (2) | | | |
| $A_0$ | 2197.11 | 2136.93 | 2070.94 | 2019.88 | 1968.01 | 2097.76 | 2027.09 | 1961.63 | 1929.09 | 1877.78 |
| $A_1$ | -1403.39 | -1356.08 | -1317.26 | -1308.93 | -1310.74 | -1544.04 | -1499.41 | -1459.13 | -1336.26 | -1291.43 |
| $A_2$ | 1449.19 | 1447.63 | 1428.56 | 1539.63 | 1614.44 | 1287.94 | 1331.56 | 1407.88 | 1493.06 | 1646.63 |
| $A_3$ | -450.26 | -515.33 | -644.89 | -599.68 | -583.61 | -518.41 | -517.04 | -501.88 | -831.89 | -924.68 |
| $R^2$ | 0.9998 | 0.9998 | 0.9997 | 0.9996 | 0.9996 | 0.9994 | 0.9994 | 0.9995 | 0.9995 | 0.9998 |
| *RMSD* | 0.016 | 0.016 | 0.017 | 0.021 | 0.019 | 0.027 | 0.025 | 0.023 | 0.022 | 0.014 |
| *%ARD* | 0.89 | 0.90 | 0.97 | 1.14 | 1.11 | 1.57 | 1.47 | 1.35 | 1.31 | 0.79 |
| | | DES3 (1) + water (2) | | | | | DES4 (1) + water (2) | | | |
| $A_0$ | 2186.81 | 2114.56 | 2034.01 | 2009.49 | 1958.05 | 2307.65 | 2227.63 | 2157.71 | 2120.71 | 2307.65 |
| $A_1$ | -1527.24 | -1458.39 | -1475.74 | -1383.16 | -1380.75 | -1459.50 | -1458.38 | -1447.00 | -1483.13 | -1459.50 |
| $A_2$ | 1537.94 | 1511.69 | 1500.44 | 1499.06 | 1478.75 | 707.25 | 756.63 | 814.38 | 822.75 | 707.25 |
| $A_3$ | -936.61 | -944.76 | -875.61 | -1011.79 | -1100.25 | -1744.50 | -1682.63 | -1697.50 | -1607.38 | -1744.50 |
| $A_4$ | - | - | - | - | - | 1861.75 | 1748.00 | 1651.56 | 1659.44 | 1861.75 |
| $R^2$ | 0.9994 | 0.9994 | 0.9991 | 0.9984 | 0.9982 | 0.9994 | 0.9992 | 0.9993 | 0.9991 | 0.9992 |

| | | | | | | | | | | |
|---|---|---|---|---|---|---|---|---|---|---|
| *RMSD* | 0.029 | 0.027 | 0.032 | 0.041 | 0.042 | 0.031 | 0.033 | 0.030 | 0.032 | 0.032 |
| *%ARD* | 1.66 | 1.54 | 1.84 | 2.24 | 2.31 | 1.63 | 1.76 | 1.62 | 1.72 | 1.72 |
| | | | | | $n_D$ | | | | | |
| | | DES1 (1) + water (2) | | | | | DES2 (1) + water (2) | | | |
| $A_0$ | 47.4950 | 47.9388 | 48.3913 | 48.7650 | 49.0938 | 45.5112 | 45.9000 | 46.3138 | 46.7975 | 47.1188 |
| $A_1$ | -36.1950 | -36.4238 | -36.6013 | -36.5350 | -36.5387 | -32.7513 | -33.5775 | -33.6663 | -34.5525 | -34.8613 |
| $A_2$ | 34.4450 | 34.8513 | 35.4238 | 35.6050 | 35.9413 | 36.2188 | 36.4450 | 36.0763 | 36.3975 | 36.1013 |
| $A_3$ | -24.9000 | -25.5913 | -26.3188 | -27.5150 | -28.4463 | -28.1588 | -27.6875 | -29.1488 | -29.4275 | -30.1938 |
| $R^2$ | 0.9996 | 0.9996 | 0.9996 | 0.9995 | 0.9995 | 0.9993 | 0.9993 | 0.9993 | 0.9994 | 0.9994 |
| *RMSD* | 0.00055 | 0.00053 | 0.00053 | 0.00056 | 0.00059 | 0.00068 | 0.00067 | 0.00065 | 0.00063 | 0.00061 |
| *%ARD* | 0.032 | 0.031 | 0.031 | 0.032 | 0.035 | 0.038 | 0.037 | 0.037 | 0.035 | 0.034 |
| | | DES3 (1) + water (2) | | | | | DES4 (1) + water (2) | | | |
| $A_0$ | 43.7650 | 44.1175 | 44.4300 | 44.8650 | 45.1475 | 45.1613 | 45.4413 | 45.7138 | 45.9388 | 46.3888 |
| $A_1$ | -34.9100 | -35.2425 | -35.5825 | -35.9075 | -36.1875 | -36.0987 | -36.3288 | -36.6013 | -36.8012 | -37.4362 |
| $A_2$ | 35.4550 | 35.4125 | 35.3850 | 35.3950 | 35.4025 | 46.8688 | 47.0938 | 47.4063 | 47.6563 | 48.1063 |
| $A_3$ | -26.8750 | -26.7625 | -26.6325 | -26.7675 | -26.9275 | -42.2863 | -43.1663 | -43.7638 | -44.2138 | -44.2988 |
| $R^2$ | 0.9993 | 0.9994 | 0.9994 | 0.9994 | 0.9994 | 0.9981 | 0.9981 | 0.9981 | 0.9981 | 0.9982 |
| *RMSD* | 0.00064 | 0.00061 | 0.00059 | 0.00059 | 0.00058 | 0.0012 | 0.0011 | 0.0011 | 0.0011 | 0.0011 |
| *%ARD* | 0.036 | 0.034 | 0.033 | 0.032 | 0.033 | 0.064 | 0.064 | 0.064 | 0.063 | 0.063 |

Table S6. Parameters $A_i$ of Eq. (6) and the corresponding *RSMD* f for TBAB:AP (DES1) + water mixtures at the temperatures (293.15 to 303.15) K and atmospheric pressure.

| Function | $T$ / K | $A_0$ | $A_1$ | $A_2$ | $A_3$ | $A_4$ | RMSD |
|---|---|---|---|---|---|---|---|
| | | | | DES1 (1) + water (2) | | | |
| | 293.15 | -3.840 | 1.863 | -0.129 | - | - | 0.018 |
| | 298.15 | -3.749 | 1.754 | -0.007 | - | - | 0.018 |
| $10^6\ V^E$ / $m^3 \cdot mol^{-1}$ | 303.15 | -3.669 | 1.631 | 0.079 | - | - | 0.018 |
| | 308.15 | -3.584 | 1.531 | 0.169 | - | - | 0.018 |
| | 313.15 | -3.499 | 1.428 | 0.223 | - | - | 0.018 |
| | 293.15 | 3.016 | -6.327 | 11.333 | -7.618 | - | 0.023 |
| | 298.15 | 2.999 | -6.024 | 10.333 | -7.210 | - | 0.019 |
| $10^4\ \Delta\alpha_p$ / $K^{-1}$ | 303.15 | 3.012 | -5.738 | 9.335 | -6.808 | - | 0.035 |
| | 308.15 | 2.930 | -5.450 | 8.342 | -6.409 | - | 0.015 |
| | 313.15 | 2.905 | -5.157 | 7.353 | -6.013 | - | 0.015 |
| | 293.15 | -2.532 | 2.831 | -2.489 | 7.174 | -7.083 | 0.0084 |
| | 298.15 | -2.451 | 2.745 | -2.487 | 6.687 | -6.168 | 0.0076 |
| $10^{10}\ k_S^E$ / $Pa^{-1}$ | 303.15 | -2.385 | 2.648 | -2.562 | 6.213 | -5.288 | 0.0074 |
| | 308.15 | -2.311 | 2.590 | -2.470 | 5.796 | -4.740 | 0.0067 |
| | 313.15 | -2.269 | 2.518 | -2.384 | 5.430 | -4.231 | 0.0056 |
| | 293.15 | 210.80 | 172.56 | 63.53 | 124.95 | - | 0.28 |
| | 298.15 | 150.86 | 115.90 | 41.61 | 86.34 | - | 0.15 |
| $\Delta\eta$ / mPa·s | 303.15 | 111.51 | 80.56 | 23.86 | 52.64 | - | 0.14 |
| | 308.15 | 83.62 | 54.50 | 26.76 | 55.38 | - | 0.09 |
| | 313.15 | 63.96 | 37.74 | 24.30 | 47.84 | - | 0.08 |
| | 293.15 | 21652 | -14563 | 13324 | - | - | 84 |
| | 298.15 | 21233 | -14378 | 13376 | - | - | 93 |
| $\Delta G^E$ / $J \cdot mol^{-1}$ | 303.15 | 20769 | -14423 | 13306 | - | - | 112 |
| | 308.15 | 20408 | -14287 | 14249 | - | - | 110 |
| | 313.15 | 20046 | -14306 | 14901 | - | - | 108 |
| | 293.15 | 0.02405 | -0.02670 | 0.01050 | - | - | 0.000098 |
| | 298.15 | 0.02333 | -0.02580 | 0.01018 | - | - | 0.000095 |
| $\Delta n_D$ | 303.15 | 0.02248 | 0.02475 | 0.01058 | - | - | 0.000081 |
| | 308.15 | 0.02158 | 0.02365 | 0.00953 | - | - | 0.000061 |
| | 313.15 | 0.02100 | 0.02250 | 0.00940 | - | - | 0.000049 |

Table S7. Parameters $A_i$ of Eq. (6) and the corresponding *RSMD* for TBAC:AP (DES2) + water mixtures at the temperatures (293.15 to 303.15) K and atmospheric pressure.

| Function | $T$ / K | $A_0$ | $A_1$ | $A_2$ | $A_3$ | $A_4$ | *RMSD* |
|---|---|---|---|---|---|---|---|
| | | | | DES2 (1) + water (2) | | | |
| | 293.15 | -3.892 | 1.937 | -0.511 | - | - | 0.013 |
| | 298.15 | -3.801 | 1.815 | -0.390 | - | - | 0.013 |
| $10^6\ V^E$ / $m^3 \cdot mol^{-1}$ | 303.15 | -3.710 | 1.706 | -0.297 | - | - | 0.012 |
| | 308.15 | -3.621 | 1.603 | -0.218 | - | - | 0.012 |
| | 313.15 | -3.533 | 1.507 | -0.147 | - | - | 0.011 |
| | 293.15 | 3.100 | -5.499 | 11.699 | -11.308 | - | 0.016 |

| | | | | | | | |
|---|---|---|---|---|---|---|---|
| | 298.15 | 3.101 | -5.451 | 10.574 | -9.668 | - | 0.011 |
| $10^4\ \Delta\alpha_p$ / $K^{-1}$ | 303.15 | 3.088 | -5.412 | 9.451 | -8.033 | - | 0.008 |
| | 308.15 | 3.088 | -5.366 | 8.333 | -6.402 | - | 0.006 |
| | 313.15 | 3.087 | -5.325 | 7.218 | -4.775 | - | 0.008 |
| | 293.15 | -2.674 | 2.973 | -2.636 | 7.238 | -7.503 | 0.0089 |
| | 298.15 | -2.587 | 2.927 | -2.676 | 6.588 | -6.704 | 0.0090 |
| $10^{10}\ k_S^E$ / $Pa^{-1}$ | 303.15 | -2.508 | 2.870 | -2.648 | 6.054 | -5.996 | 0.0083 |
| | 308.15 | -2.426 | 2.822 | -2.610 | 5.580 | -5.427 | 0.0073 |
| | 313.15 | -2.379 | 2.757 | -2.545 | 5.143 | -4.959 | 0.0068 |
| | 293.15 | 186.34 | 107.75 | 7.62 | 119.36 | - | 0.48 |
| | 298.15 | 131.40 | 69.93 | 12.51 | 91.90 | - | 0.31 |
| $\Delta\eta$ / mPa·s | 303.15 | 96.61 | 47.32 | 17.87 | 76.02 | - | 0.21 |
| | 308.15 | 74.69 | 40.07 | 14.30 | 42.15 | - | 0.10 |
| | 313.15 | 57.16 | 30.35 | 18.05 | 31.69 | - | 0.06 |
| | 293.15 | 20791 | -15851 | 11984 | - | - | 105 |
| | 298.15 | 20268 | -15531 | 12383 | - | - | 104 |
| $\Delta G^E$ / J·mol$^{-1}$ | 303.15 | 19795 | -15202 | 13049 | - | - | 100 |
| | 308.15 | 19622 | -15047 | 13933 | - | - | 141 |
| | 313.15 | 19271 | -14950 | 15285 | - | - | 147 |
| | 293.15 | 0.02351 | 0.02009 | 0.02854 | 0.01186 | - | 0.000056 |
| | 298.15 | 0.02213 | 0.02198 | 0.02683 | 0.00743 | - | 0.000062 |
| $\Delta n_D$ | 303.15 | 0.02174 | 0.02009 | 0.02271 | 0.01261 | - | 0.000045 |
| | 308.15 | 0.02006 | 0.02169 | 0.02104 | 0.01136 | - | 0.000061 |
| | 313.15 | 0.01940 | 0.02135 | 0.01775 | 0.01340 | - | 0.000050 |

Table S8 Parameters $A_i$ of Eq. (6) and the corresponding *RSMD* for TBAB:MAE (DES3) + water mixtures at the temperatures (293.15 to 303.15) K and atmospheric pressure.

| Function | $T$ / K | $A_0$ | $A_1$ | $A_2$ | $A_3$ | $A_4$ | *RMSD* |
|---|---|---|---|---|---|---|---|
| | | | | DES3 (1) + water (2) | | | |
| | 293.15 | -4.274 | 2.720 | -1.044 | - | - | 0.011 |
| | 298.15 | -4.200 | 2.580 | -0.924 | - | - | 0.011 |
| $10^6\ V^E$ / m$^3$·mol$^{-1}$ | 303.15 | -4.126 | 2.452 | -0.816 | - | - | 0.010 |
| | 308.15 | -4.051 | 2.335 | -0.717 | - | - | 0.010 |
| | 313.15 | -3.977 | 2.221 | -0.633 | - | - | 0.010 |
| | 293.15 | 2.765 | -5.444 | 7.854 | -12.486 | 8.919 | 0.015 |
| | 298.15 | 2.732 | -5.304 | 7.553 | -11.321 | 7.769 | 0.014 |
| $10^4\ \Delta\alpha_p$ / $K^{-1}$ | 303.15 | 2.696 | -5.166 | 7.256 | -10.159 | 6.625 | 0.015 |
| | 308.15 | 2.672 | -5.024 | 6.963 | -9.000 | 5.485 | 0.014 |
| | 313.15 | 2.642 | -4.891 | 6.668 | -7.846 | 4.348 | 0.014 |
| | 293.15 | -3.164 | 3.551 | -2.920 | 8.644 | -9.214 | 0.0120 |
| | 298.15 | -3.106 | 3.531 | -2.911 | 8.059 | -8.443 | 0.0108 |
| $10^{10}\ k_S^E$ / $Pa^{-1}$ | 303.15 | -3.070 | 3.505 | -2.864 | 7.535 | -7.827 | 0.0097 |
| | 308.15 | -3.036 | 3.489 | -2.846 | 7.071 | -7.230 | 0.0088 |
| | 313.15 | -3.018 | 3.446 | -2.814 | 6.704 | -6.730 | 0.0082 |
| $\Delta\eta$ / mPa·s | 293.15 | 155.32 | 50.59 | -29.09 | 71.03 | - | 0.16 |

| | | | | | | | |
|---|---|---|---|---|---|---|---|
| | 298.15 | 111.72 | 35.88 | -17.09 | 48.62 | - | 0.10 |
| | 303.15 | 82.12 | 18.82 | -11.04 | 44.44 | - | 0.11 |
| | 308.15 | 64.46 | 16.44 | -8.22 | 29.55 | - | 0.11 |
| | 313.15 | 50.02 | 10.03 | -7.77 | 20.57 | - | 0.09 |
| | 293.15 | 21711 | -16843 | 14212 | - | - | 157 |
| | 298.15 | 21183 | -16342 | 14037 | - | - | 157 |
| $\Delta G^E$ / J·mol$^{-1}$ | 303.15 | 20579 | -16359 | 13958 | - | - | 155 |
| | 308.15 | 20452 | -15983 | 14035 | - | - | 183 |
| | 313.15 | 20103 | -16232 | 13937 | - | - | 196 |
| | 293.15 | 0.02393 | -0.03685 | 0.02643 | - | - | 0.000023 |
| | 298.15 | 0.02318 | -0.03580 | 0.02423 | - | - | 0.000020 |
| $\Delta n_D$ | 303.15 | 0.02245 | -0.03500 | 0.02230 | - | - | 0.000027 |
| | 308.15 | 0.02163 | -0.03415 | 0.02023 | - | - | 0.000024 |
| | 313.15 | 0.02088 | -0.03335 | 0.01863 | - | - | 0.000035 |

Table S9 Parameters $A_i$ of Eq. (6) and the corresponding *RSMD* for TBAB:BAE (DES4) + water mixtures at the temperatures (293.15 to 303.15) K and atmospheric pressure.

| Function | $T$ / K | $A_0$ | $A_1$ | $A_2$ | $A_3$ | $A_4$ | *RMSD* |
|---|---|---|---|---|---|---|---|
| | | | DES4 (1) + water (2) | | | | |
| | 293.15 | -4.045 | 2.112 | -0.822 | - | - | 0.019 |
| | 298.15 | -3.954 | 1.994 | -0.710 | - | - | 0.017 |
| $10^6$ $V^E$ / $m^3 \cdot mol^{-1}$ | 303.15 | -3.860 | 1.884 | -0.621 | - | - | 0.016 |
| | 308.15 | -3.763 | 1.779 | -0.548 | - | - | 0.015 |
| | 313.15 | -3.662 | 1.680 | -0.490 | - | - | 0.014 |
| | 293.15 | 2.570 | -3.022 | 2.439 | -13.078 | 15.448 | 0.026 |
| | 298.15 | 2.615 | -3.185 | 2.404 | -11.655 | 13.894 | 0.025 |
| $10^4$ $\Delta\alpha_p$ / $K^{-1}$ | 303.15 | 2.632 | -3.385 | 2.349 | -10.245 | 12.347 | 0.025 |
| | 308.15 | 2.710 | -3.521 | 2.324 | -8.834 | 10.806 | 0.025 |
| | 313.15 | 3.299 | -4.296 | 2.684 | -9.634 | 11.835 | 0.030 |
| | 293.15 | -2.469 | 2.659 | -1.919 | 7.471 | -8.403 | 0.0110 |
| | 298.15 | -2.444 | 2.599 | -1.975 | 7.146 | -7.802 | 0.0100 |
| $10^{10}$ $k_S^E$ / $Pa^{-1}$ | 303.15 | -2.436 | 2.580 | -1.805 | 6.840 | -7.601 | 0.0094 |
| | 308.15 | -2.391 | 2.544 | -1.838 | 6.581 | -7.193 | 0.0083 |
| | 313.15 | -2.366 | 2.535 | -1.999 | 6.310 | -6.681 | 0.0086 |
| | 293.15 | 190.38 | 80.90 | -55.30 | 24.71 | - | 0.18 |
| | 298.15 | 135.50 | 48.85 | -35.76 | 24.08 | - | 0.14 |
| $\Delta\eta$ / mPa·s | 303.15 | 100.35 | 30.76 | -24.54 | 17.62 | - | 0.09 |
| | 308.15 | 76.61 | 17.27 | -15.40 | 17.10 | - | 0.08 |
| | 313.15 | 59.55 | 6.93 | -12.38 | 22.52 | - | 0.07 |
| | 293.15 | 23563 | -19656 | 16934 | - | - | 299 |
| | 298.15 | 23008 | -19563 | 16883 | - | - | 291 |
| $\Delta G^E$/ $J \cdot kmol^{-1}$ | 303.15 | 22545 | -19569 | 17015 | - | - | 289 |
| | 308.15 | 22329 | -19709 | 17139 | - | - | 281 |
| | 313.15 | 22100 | -19957 | 17170 | - | - | 260 |
| | 293.15 | 0.02000 | -0.02513 | 0.02695 | - | - | 0.000029 |
| | 298.15 | 0.01929 | -0.02399 | 0.02551 | - | - | 0.000027 |
| $\Delta n_D$ | 303.15 | 0.01854 | -0.02324 | 0.02481 | - | - | 0.000024 |
| | 308.15 | 0.01773 | -0.02273 | 0.02413 | - | - | 0.000029 |
| | 313.15 | 0.01686 | -0.02321 | 0.02319 | - | - | 0.000020 |

Table S10 Isobaric thermal expansion coefficient ($\alpha_p$), isochoric molar heat capacity($C_P$ ), Flory theory parameters: characteristic volume($V$*), reduce volume ($\tilde{V}$ ), characteristic pressure ($P$*), and ratio of molecular surface to volume ratio ($S_1/S_2$) of DES to water.

| DES | $T$ / K | $10^4\, \alpha$ / $K^{-1}$ | $C_p$ / $J \cdot mol^{-1} \cdot K^{-1}$ | $10^6\, V^*$ / $m^{-3} \cdot mol^{-1}$ | $\tilde{V}$ | $10^8 P^*$ / Pa | $S_1/S_2$ |
|---|---|---|---|---|---|---|---|
| DES1 | | | | | | | |
| | 293.15 | 7.334 | 257.4 | 91.563 | 1.188 | 7.199 | 0.571 |
| | 298.15 | 7.343 | 260.7 | 91.670 | 1.191 | 7.209 | 0.569 |
| | 303.15 | 7.351 | 264.0 | 91.780 | 1.194 | 7.215 | 0.566 |
| | 308.15 | 7.359 | 268.4 | 91.890 | 1.196 | 7.221 | 0.564 |
| | 313.15 | 7.368 | 272.8 | 92.003 | 1.199 | 7.226 | 0.562 |
| DES2 | | | | | | | |
| | 293.15 | 7.305 | 249.8 | 90.515 | 1.187 | 7.111 | 0.573 |
| | 298.15 | 7.315 | 250.9 | 90.618 | 1.190 | 7.110 | 0.571 |
| | 303.15 | 7.325 | 252.9 | 90.721 | 1.193 | 7.115 | 0.568 |
| | 308.15 | 7.336 | 254.0 | 90.827 | 1.196 | 7.110 | 0.566 |
| | 313.15 | 7.346 | 256.1 | 90.934 | 1.199 | 7.108 | 0.564 |
| DES3 | | | | | | | |
| | 293.15 | 7.342 | 187.7 | 94.326 | 1.188 | 5.550 | 0.565 |
| | 298.15 | 7.372 | 190.7 | 94.399 | 1.191 | 5.574 | 0.563 |
| | 303.15 | 7.403 | 194.7 | 94.473 | 1.195 | 5.601 | 0.561 |
| | 308.15 | 7.433 | 203.2 | 94.549 | 1.198 | 5.646 | 0.559 |
| | 313.15 | 7.463 | 212.4 | 94.627 | 1.202 | 5.690 | 0.557 |
| DES4 | | | | | | | |
| | 293.15 | 7.725 | 284.2 | 130.676 | 1.196 | 5.255 | 0.507 |
| | 298.15 | 7.754 | 288.2 | 130.796 | 1.200 | 5.266 | 0.505 |
| | 303.15 | 7.783 | 290.6 | 130.918 | 1.203 | 5.270 | 0.503 |
| | 308.15 | 7.813 | 294.0 | 131.044 | 1.207 | 5.277 | 0.501 |
| | 313.15 | 7.842 | 297.9 | 131.173 | 1.210 | 5.282 | 0.499 |
| Water | | | | | | | |
| | 293.15 | 2.120 | 75.4 | 17.031 | 1.060 | 1.520 | - |
| | 298.15 | 2.565 | 75.3 | 16.843 | 1.073 | 1.945 | - |
| | 303.15 | 3.010 | 75.3 | 16.661 | 1.086 | 2.405 | - |
| | 308.15 | 3.455 | 75.2 | 16.485 | 1.099 | 2.898 | - |
| | 313.15 | 3.901 | 75.2 | 16.315 | 1.113 | 3.420 | - |

Table S11 Partial molar volumes of DESs and water in their binary mixtures at $T$ = (293.15 to 313.15) K and at atmospheric pressure (0.1 MPa)[a]

| $x_1$ | $10^6 \bar{V}_1$ / $m^3 \cdot mol^{-1}$ | $10^6 \bar{V}_2$ / $m^3 \cdot mol^{-1}$ | $10^6 \bar{V}_1$ / $m^3 \cdot mol^{-1}$ | $10^6 \bar{V}_2$ / $m^3 \cdot mol^{-1}$ | $10^6 \bar{V}_1$ / $m^3 \cdot mol^{-1}$ | $10^6 \bar{V}_2$ / $m^3 \cdot mol^{-1}$ | $10^6 \bar{V}_1$ / $m^3 \cdot mol^{-1}$ | $10^6 \bar{V}_2$ / $m^3 \cdot mol^{-1}$ | $10^6 \bar{V}_1$ / $m^3 \cdot mol^{-1}$ | $10^6 \bar{V}_2$ / $m^3 \cdot mol^{-1}$ |
|---|---|---|---|---|---|---|---|---|---|---|
| $T$ / K | 293.15 | | 298.15 | | 303.15 | | 308.15 | | 313.15 | |
| DES1 (1) + water (2) | | | | | | | | | | |
| 0.0000 | 102.91 | 18.05 | 103.60 | 18.07 | 104.32 | 18.09 | 105.00 | 18.12 | 105.65 | 18.16 |
| 0.0994 | 104.68 | 18.03 | 105.20 | 18.05 | 105.79 | 18.07 | 106.33 | 18.10 | 106.87 | 18.13 |
| 0.1981 | 106.03 | 17.98 | 106.45 | 17.98 | 106.96 | 18.01 | 107.42 | 18.03 | 107.89 | 18.06 |
| 0.2979 | 107.04 | 17.88 | 107.41 | 17.88 | 107.87 | 17.91 | 108.30 | 17.93 | 108.73 | 17.95 |
| 0.3961 | 107.75 | 17.75 | 108.10 | 17.73 | 108.54 | 17.77 | 108.95 | 17.79 | 109.36 | 17.82 |
| 0.4983 | 108.24 | 17.56 | 108.59 | 17.53 | 109.03 | 17.59 | 109.43 | 17.61 | 109.83 | 17.64 |
| 0.5987 | 108.53 | 17.32 | 108.90 | 17.29 | 109.32 | 17.37 | 109.73 | 17.41 | 110.13 | 17.44 |
| 0.6999 | 108.68 | 17.04 | 109.06 | 17.00 | 109.49 | 17.12 | 109.89 | 17.17 | 110.29 | 17.21 |
| 0.8000 | 108.74 | 16.71 | 109.13 | 16.67 | 109.55 | 16.83 | 109.95 | 16.90 | 110.36 | 16.95 |
| 0.9000 | 108.75 | 16.35 | 109.14 | 16.30 | 109.55 | 16.50 | 109.96 | 16.59 | 110.36 | 16.65 |
| 1.0000 | 108.74 | 15.94 | 109.14 | 15.88 | 109.54 | 16.13 | 109.95 | 16.24 | 110.35 | 16.31 |
| $V_i^o$ | 108.74 | 18.05 | 109.14 | 18.07 | 109.54 | 18.09 | 109.95 | 18.12 | 110.35 | 18.16 |
| DES2 (1) + water (2) | | | | | | | | | | |
| 0.0000 | 101.23 | 18.05 | 101.94 | 18.07 | 102.63 | 18.09 | 103.30 | 18.12 | 103.95 | 18.16 |
| 0.0986 | 103.22 | 18.04 | 103.78 | 18.06 | 104.32 | 18.08 | 104.87 | 18.11 | 105.40 | 18.14 |
| 0.1973 | 104.69 | 17.99 | 105.15 | 18.01 | 105.62 | 18.03 | 106.08 | 18.05 | 106.54 | 18.08 |
| 0.2961 | 105.74 | 17.91 | 106.15 | 17.92 | 106.57 | 17.93 | 106.99 | 17.95 | 107.41 | 17.98 |
| 0.3962 | 106.46 | 17.76 | 106.86 | 17.77 | 107.25 | 17.79 | 107.65 | 17.81 | 108.05 | 17.84 |
| 0.4925 | 106.91 | 17.57 | 107.30 | 17.58 | 107.69 | 17.60 | 108.08 | 17.63 | 108.48 | 17.66 |
| 0.5954 | 107.20 | 17.29 | 107.59 | 17.32 | 107.98 | 17.35 | 108.38 | 17.38 | 108.78 | 17.42 |
| 0.6946 | 107.34 | 16.97 | 107.74 | 17.01 | 108.14 | 17.05 | 108.53 | 17.1 | 108.93 | 17.15 |
| 0.7996 | 107.41 | 16.56 | 107.81 | 16.62 | 108.21 | 16.69 | 108.61 | 16.75 | 109.01 | 16.82 |
| 0.9019 | 107.43 | 16.12 | 107.83 | 16.20 | 108.23 | 16.28 | 108.62 | 16.37 | 109.02 | 16.45 |
| 1.0000 | 107.43 | 15.66 | 107.83 | 15.76 | 108.23 | 15.86 | 108.62 | 15.96 | 109.02 | 16.05 |
| $V_i^o$ | 107.44 | 18.05 | 107.83 | 18.07 | 108.23 | 18.09 | 108.62 | 18.12 | 109.02 | 18.16 |
| DES3 (1) + water (2) | | | | | | | | | | |
| 0.0000 | 104.00 | 18.05 | 104.75 | 18.07 | 105.47 | 18.09 | 106.18 | 18.12 | 106.88 | 18.16 |

| | | | | | | | | | | |
|---|---|---|---|---|---|---|---|---|---|---|
| 0.0978 | 106.93 | 18.06 | 107.51 | 18.07 | 108.07 | 18.10 | 108.64 | 18.12 | 109.20 | 18.15 |
| 0.1982 | 109.00 | 18.05 | 109.48 | 18.06 | 109.95 | 18.08 | 110.42 | 18.10 | 110.90 | 18.13 |
| 0.2957 | 110.33 | 18.00 | 110.75 | 18.01 | 111.17 | 18.02 | 111.60 | 18.03 | 112.04 | 18.06 |
| 0.3970 | 111.17 | 17.88 | 111.57 | 17.88 | 111.98 | 17.89 | 112.39 | 17.90 | 112.80 | 17.92 |
| 0.4958 | 111.64 | 17.67 | 112.03 | 17.67 | 112.43 | 17.69 | 112.84 | 17.70 | 113.26 | 17.73 |
| 0.5935 | 111.87 | 17.38 | 112.27 | 17.39 | 112.68 | 17.41 | 113.09 | 17.43 | 113.51 | 17.46 |
| 0.6956 | 111.97 | 16.98 | 112.38 | 17.00 | 112.80 | 17.04 | 113.21 | 17.07 | 113.63 | 17.11 |
| 0.7979 | 112.01 | 16.50 | 112.43 | 16.55 | 112.84 | 16.59 | 113.26 | 16.65 | 113.69 | 16.70 |
| 0.8940 | 112.03 | 16.01 | 112.44 | 16.07 | 112.86 | 16.13 | 113.28 | 16.20 | 113.70 | 16.27 |
| 1.000 | 112.04 | 15.45 | 112.45 | 15.52 | 112.87 | 15.60 | 113.29 | 15.69 | 113.71 | 15.77 |
| $V_i^o$ | 112.04 | 18.05 | 112.45 | 18.07 | 112.87 | 18.09 | 113.29 | 18.12 | 113.71 | 18.16 |
| DES4 (1) + water (2) | | | | | | | | | | |
| 0.0000 | 149.34 | 18.05 | 150.27 | 18.07 | 151.17 | 18.09 | 152.06 | 18.12 | 152.94 | 18.16 |
| 0.0975 | 151.75 | 18.05 | 152.52 | 18.07 | 153.29 | 18.09 | 154.05 | 18.12 | 154.82 | 18.15 |
| 0.1951 | 153.45 | 18.02 | 154.13 | 18.04 | 154.81 | 18.06 | 155.50 | 18.08 | 156.19 | 18.11 |
| 0.2936 | 154.61 | 17.95 | 155.24 | 17.96 | 155.87 | 17.98 | 156.51 | 18.00 | 157.17 | 18.03 |
| 0.3943 | 155.37 | 17.81 | 155.97 | 17.82 | 156.58 | 17.83 | 157.21 | 17.85 | 157.84 | 17.88 |
| 0.4930 | 155.81 | 17.58 | 156.41 | 17.60 | 157.02 | 17.62 | 157.63 | 17.64 | 158.25 | 17.68 |
| 0.5925 | 156.06 | 17.27 | 156.67 | 17.30 | 157.27 | 17.33 | 157.89 | 17.36 | 158.51 | 17.40 |
| 0.6975 | 156.20 | 16.85 | 156.81 | 16.90 | 157.42 | 16.94 | 158.03 | 16.99 | 158.66 | 17.04 |
| 0.7920 | 156.26 | 16.41 | 156.87 | 16.47 | 157.49 | 16.53 | 158.10 | 16.59 | 158.72 | 16.66 |
| 0.8938 | 156.30 | 15.88 | 156.91 | 15.96 | 157.52 | 16.04 | 158.14 | 16.12 | 158.76 | 16.20 |
| 1.0000 | 156.32 | 15.29 | 156.93 | 15.40 | 157.54 | 15.50 | 158.15 | 15.59 | 158.77 | 15.68 |
| $V_i^o$ | 156.32 | 18.05 | 156.93 | 18.07 | 157.54 | 18.09 | 158.15 | 18.12 | 158.77 | 18.16 |
